# The effect of exercise intervention on atherosclerosis prevention in overweight or obese adults: A Bayesian network meta-analysis of randomized controlled trials

**DOI:** 10.1371/journal.pone.0344674

**Published:** 2026-03-13

**Authors:** ChunBaiXue Yang, Yang Xu, XiuPeng Li, HangLin Yu, MingRui Wang, Bing Yang

**Affiliations:** 1 Physical Education College, Bohai University, Jinzhou, PR China; 2 Physical Education College, Southwest University, Chongqing, PR China; 3 School of Sport Science, Beijing Sport University, Beijing, PR China; Japanese Academy of Health and Practice, JAPAN

## Abstract

**Background:**

Obesity is a major modifiable risk factor for atherosclerotic cardiovascular disease. This study evaluated the efficacy of different exercise modalities in improving vascular health parameters in overweight/obese adults.

**Methods:**

We conducted a systematic review and network meta-analysis of 51 randomized controlled trials (n = 2638) following PRISMA and a prospectively registered PROSPERO protocol (CRD420251066443). PubMed, Web of Science, the Cochrane Library, and EBSCO SPORTDiscus were searched from inception to 31 May 2025, supplemented by Google Scholar and reference screening. Eligible trials enrolled adults aged 18–65 years with overweight/obesity, included interventions lasting ≥4 weeks, and compared CET, RT, INT, CT, or HYB with usual lifestyle/standard care. The primary outcome was FMD, with CIMT and PWV as secondary outcomes. Risk of bias was assessed using RoB 2 and certainty of evidence using CINeMA. Effects were synthesized as SMDs with 95% CIs, and modalities were compared using network meta-analysis.

**Results:**

The certainty of evidence was assessed using CINeMA, with a low confidence rating. Fifty-one trials (n = 2,638) met the inclusion criteria. Compared to the control group, exercise interventions led to significant improvements in Flow-Mediated Dilation (FMD) (SMD = 0.99, 95% CI 0.69–1.29), reductions in Pulse Wave Velocity (PWV) (SMD = −0.31, 95% CI −0.44 to −0.18), and decreases in Carotid Intima-Media Thickness (CIMT) (SMD = −0.20, 95% CI −0.36 to −0.05). Network meta-analysis revealed that HYB was most effective for improving FMD, INT was most effective for reducing PWV, and CET and RT exhibited similar effects on CIMT. Subgroup analyses indicated that the effects were more pronounced in women and in Asian populations.

**Conclusions:**

Exercise interventions improve vascular health in overweight/obese adults, with modality-specific patterns across vascular domains. HYB appears most favorable for enhancing endothelial function (FMD), INT may offer greater benefits for reducing arterial stiffness (PWV), and both CET and RT show comparable effects on structural remodeling (CIMT).

## Introduction

Obesity and overweight have become among the most critical public health concerns worldwide in the 21st century [1]. According to data from the World Health Organization (WHO), the global prevalence of obesity has nearly tripled since 1975. As of 2016, over 1.9 billion adults were classified as overweight, with more than 650 million meeting the criteria for obesity (body mass index [BMI] ≥ 30 kg/m²) [2–4]. Obesity is strongly associated not only with metabolic syndrome, type 2 diabetes, and hypertension, but also represents a major modifiable risk factor for atherosclerotic cardiovascular disease (ASCVD) [5–7]. Atherosclerosis, the principal pathological basis of cardiovascular conditions such as coronary artery disease and stroke, is closely linked to obesity-related disturbances in lipid metabolism, chronic low-grade inflammation, insulin resistance, and endothelial dysfunction [8–11].

Atherosclerosis is now widely recognized as a chronic inflammatory condition characterized by endothelial injury, lipid accumulation, foam cell formation, and progressive plaque development [12]. Individuals with obesity commonly exhibit lipid abnormalities—including elevated low-density lipoprotein cholesterol (LDL-C) and reduced high-density lipoprotein cholesterol (HDL-C)—alongside insulin resistance, increased oxidative stress, and heightened levels of pro-inflammatory cytokines such as tumour necrosis factor-alpha (TNF-α) and interleukin-6 (IL-6) [13]. These factors collectively accelerate the initiation and progression of atherosclerotic lesions. In addition, visceral adiposity contributes to adipose tissue dysfunction [14], leading to increased release of free fatty acids and adipokines, which further exacerbate vascular inflammation and impair endothelial function [15–18].

Exercise intervention is widely recommended as a first-line non-pharmacological strategy for both the prevention and management of obesity and cardiovascular disease [19]. Accumulating evidence supports the multifaceted benefits of regular physical activity on metabolic and vascular health. These include: (1) reductions in total and visceral fat mass [20]; (2) improvements in lipid profiles (e.g., decreased LDL-C and triglycerides, increased HDL-C) [21]; (3) enhanced insulin sensitivity [22]; (4) decreased levels of systemic inflammatory markers such as C-reactive protein [23]; and (5) improved endothelial function through increased nitric oxide (NO)-mediated vasodilation [24]. Furthermore, exercise has been shown to modulate autonomic function, lower blood pressure, and improve myocardial perfusion, thereby offering additional protection against atherosclerotic progression [25].

In recent years, multiple randomised controlled trials (RCTs) and observational studies have investigated the effects of exercise on surrogate markers of atherosclerosis in overweight and obese populations. Commonly assessed outcomes include carotid intima-media thickness (CIMT), arterial stiffness (measured by pulse wave velocity, PWV), endothelial function (assessed by flow-mediated dilation, FMD), and atherosclerotic plaque burden [26]. Both aerobic exercise modalities and resistance training have been reported to produce significant improvements in CIMT and vascular compliance, while high-intensity interval training (HIIT) may offer greater enhancements in endothelial function [27–29]. However, findings across studies remain heterogeneous, potentially due to variations in exercise type, intensity, and duration, as well as differences in participants’ baseline characteristics and follow-up durations.

While previous meta-analyses have explored the effects of exercise on conventional cardiovascular risk factors such as blood lipids, glucose levels, and blood pressure, systematic reviews specifically targeting atherosclerotic outcomes in overweight or obese individuals remain limited. Many existing reviews focus on single endpoints such as CIMT or FMD and fail to capture the broader impact of exercise on atherosclerotic progression. Moreover, key uncertainties persist regarding the optimal type and dosage of exercise, the relative efficacy of aerobic versus resistance or combined training, and the role of long-term adherence in mediating vascular outcomes. Therefore, a comprehensive and methodologically robust meta-analysis is warranted to synthesise current evidence and inform clinical and public health recommendations.

## Materials and methods

### Protocol and registration

This systematic review and network meta-analysis was conducted in accordance with the Preferred Reporting Items for Systematic Reviews and Meta-Analyses (PRISMA) guideline [30]. The study protocol was prospectively registered in the International Prospective Register of Systematic Reviews (PROSPERO; Registration No. CRD420251066443). The full protocol is available at: https://www.crd.york.ac.uk/prospero/display_record.php?ID=CRD420251066443.

### Search strategy

A comprehensive literature search was performed in four electronic databases (PubMed, Web of Science, the Cochrane Library, and EBSCO SPORTDiscus) from inception through 31May 2025. The search targeted terms in titles, abstracts, and keywords, and combined controlled vocabulary (when applicable) with free-text terms for population and intervention concepts. Briefly, the search combined synonyms for overweight/obesity and adults with exercise-related terms (e.g., exercise, aerobic/endurance training, resistance/strength training, interval training, combined training, and hybrid/mixed-modality programs) and vascular/atherosclerosis-related terms (e.g., atherosclerosis, arterial stiffness, carotid atherosclerosis, and cardiovascular health). To maximize sensitivity and avoid missing studies that reported vascular outcomes using different terminology, outcome concepts were not used as restrictive filters. In addition, we performed manual screening of Google Scholar and the reference lists of relevant articles to identify additional eligible studies. Full database-specific search strategies are provided in the Supplementary Materials (S1 Table).

### Eligibility criteria

Eligible interventions were structured exercise programs classified as continuous endurance/aerobic training, resistance/strength training, interval training, combined training (aerobic plus resistance), or hybrid/recreational mixed-modality programs. Comparators were usual lifestyle or standard care. Trials were required to report sufficient post-intervention outcome data for quantitative synthesis in at least one prespecified vascular outcome, with flow-mediated dilation (FMD), carotid intima-media thickness (CIMT), and pulse-wave velocity (PWV) considered the primary outcomes. We included interventions lasting at least 4 weeks and excluded studies with non-randomized designs, ineligible populations, or insufficient outcome data for quantitative synthesis.

### Study selection

Two reviewers independently screened titles, abstracts and full texts in a double-blind manner; disagreements were settled by a third reviewer. Duplicates were removed with EndNote 19. Screening proceeded hierarchically—from title to abstract to full text—until the final study set was established.

### Data extraction and synthesis

For each eligible study we extracted: first author, publication year, country, sample size (intervention vs control), exercise modality, intervention length and frequency, outcomes of interest and adverse events. Eligible exercise programmes were classified into five categories (CET, INT, RT, CT, and HYB) using prespecified operational criteria (frequency, intensity thresholds, and session duration), as detailed in Supplementary Materials (S2 Table). Two reviewers independently assigned each intervention to a category based on the trial descriptions; discrepancies were resolved by consensus.

Outcome data were extracted as post-intervention mean ± SD for each group and pooled as standardised mean differences (SMDs). We prioritised post-intervention values because many trials did not report SDs of change scores or sufficient information (e.g., baseline–follow-up correlations) to derive them reliably. When numerical data were missing, corresponding authors were contacted; failing that, values were digitised with GetData Graph Digitizer.

When SDs were not reported but other summary statistics were available, SDs were derived as follows:


$$SD=SE\times \sqrt{N}\;$$

$$SE=\frac{{CI}_{\text{h}ig\text{h}}-{CI}_{low}}{\text{2}\times t_{N-\text{1},\text{1}-\alpha /\text{2}}},SD=SE\times \sqrt{N}$$

where N is the sample size of the corresponding group, $\;{CI}_{low}$ and ${CI}_{\text{h}ig\text{h}}$ are the lower and upper limits of the confidence interval for the mean, α is the significance level (for a 95% CI, α=0.05), and $t_{N-\text{1},\text{1}-\alpha /\text{2}}$ is the critical value from the t-distribution with N-1 degrees of freedom. The full SD derivation workflow and the list of trials requiring conversion are provided in S2 File.

### Risk of bias assessment

The included studies were assessed using the Cochrane Risk of Bias 2 tool. Two reviewers independently evaluated risk of bias in accordance with the quality assessment criteria recommended in the Cochrane Handbook (version 5.3) across five domains: bias arising from the randomization process, bias due to deviations from intended interventions, bias due to missing outcome data, bias in measurement of the outcome, and bias in selection of the reported result. Any disagreements were resolved through discussion with a third reviewer.

### GRADE assessments

Certainty of evidence was evaluated using the GRADE approach. Because this review synthesized evidence using network meta-analysis, GRADE assessments were implemented through the CINeMA framework, which operationalizes GRADE for network estimates across six domains (within-study bias, reporting bias, indirectness, imprecision, heterogeneity, and incoherence) [31]. For each comparison, evidence was rated as having no concerns, some concerns (downgraded by one level), or major concerns (downgraded by two levels), and an overall certainty rating (high, moderate, low, or very low) was assigned and considered when interpreting relative effects and treatment rankings.

### Statistical analysis

A network meta-analysis was conducted in R. Full model equations, priors, and implementation details (including software environment and MCMC settings) are provided in S3 File. Continuous outcomes were pooled as standardised mean differences (SMDs) with 95% confidence intervals (CIs).The transitivity assumption was assessed by comparing the distributions of key effect modifiers across comparisons and nodes, including age, proportion of women, BMI, and baseline values of the corresponding vascular outcome (FMD, PWV, or CIMT), where reported (S3 Table). Standardized mean differences (SMDs) were calculated using post-intervention means and standard deviations for each group; thus, no assumptions about pre–post correlations were required for the primary analyses. For a small subset of trials in which change-score SDs were required, we imputed a pre–post correlation of r = 0.50 and conducted sensitivity analyses using r values from 0.30 to 0.70; results were materially unchanged.Statistical heterogeneity was quantified with I²: < 50% (low), 50–75% (moderate), > 75% (high). Effect magnitudes were interpreted as trivial (SMD ≤ 0.20), small (0.20 < SMD < 0.50), moderate (0.50 ≤ SMD < 0.80) or large (SMD ≥ 0.80). Potential publication bias and small‐study effects were explored using funnel plots. Posterior distributions were examined using kernel density plots. Markov‐chain convergence was assessed visually with trace plots and quantified using the Gelman–Rubin statistic (R^; R-hat) and effective sample sizes (ESS) for all model parameters.(S4 Table) Convergence was deemed adequate when R^ values were close to 1.00 and ESS indicated stable estimation. MCMC sampling was performed using four chains with a prespecified warm-up/burn-in and no thinning. To assess sensitivity to Monte Carlo error, we increased the number of iterations beyond 7,000 and confirmed that posterior summaries and treatment rankings were stable. Meta-regression analyses were conducted to examine potential sources of heterogeneity, and network geometry was visualised using MetaInsight. Sensitivity analyses were performed by stratifying studies according to PWV segment (central vs peripheral) and repeating analyses after excluding studies with unspecified PWV segments.Treatment ranking and uncertainty. Treatment rankings were summarised using posterior rank probabilities and SUCRA (0–1) derived from the Bayesian random-effects NMA. For each posterior draw, treatments were ranked based on the relative effect estimates; rank probabilities and cumulative ranking curves were then obtained across draws, and SUCRA was calculated as the area under the cumulative ranking curve for each modality and outcome (S1 Fig).

## Results

### Study selection

**Fig 1:**
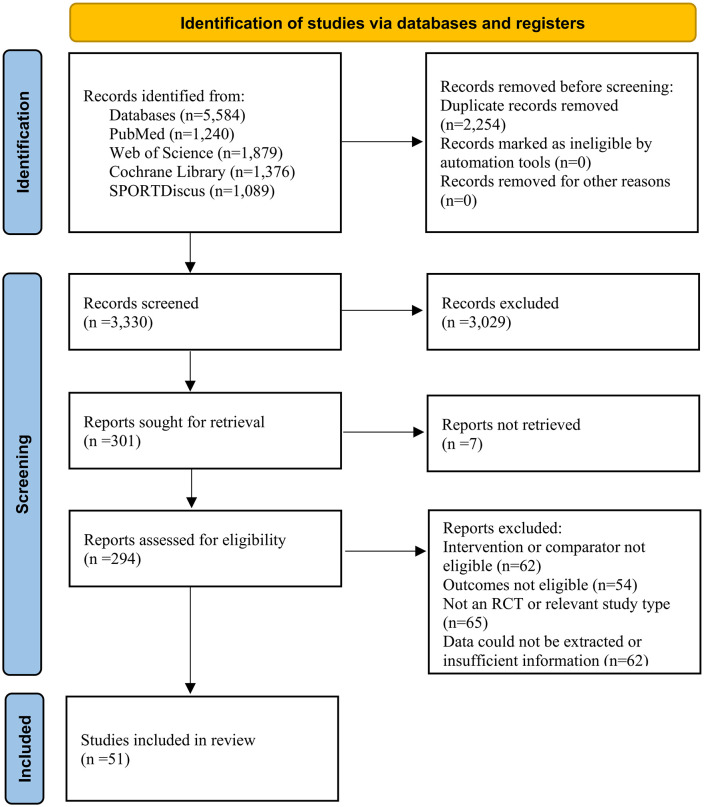
Flowchart of study selection for inclusion trials in the systematic review.

The database search yielded 5584 records. After duplicate removal, 3350 unique articles remained. Title/abstract screening excluded non-exercise studies, leaving 294 full texts. Following full-text review—excluding studies with ineligible interventions or comparators, non-RCT designs, ineligible outcomes, or insufficient data—51 RCTs met all criteria (Fig 1).

### Study-quality assessment

In accordance with the quality assessment criteria recommended in the Cochrane Handbook (version 5.3), the risk of bias was evaluated for the 51 included randomized controlled trials (RCTs). The results indicated that all included studies had some degree of bias. Detailed risk-of-bias assessments are provided in S1 File.

### GRADE assessments

As shown in Table 1, CINeMA assessments indicated that within-study bias was consistently rated as some concerns across all control–intervention comparisons, whereas reporting bias was generally judged as low risk. Indirectness was rated as no concerns for CET, CT, and RT, but some concerns for HYB and INT. For imprecision, major concerns were observed for HYB and RT, while CT and INT showed some concerns and CET showed no concerns. Heterogeneity raised major concerns for CET and some concerns for CT and INT, while no concerns were noted for HYB and RT. Formal inconsistency diagnostics (node-splitting/design-by-treatment interaction) were not estimable because the network contained few closed loops; therefore, inconsistency could not be quantified for this outcome network. Accordingly, we did not base certainty judgments on statistical inconsistency tests and interpreted treatment rankings cautiously. Overall, these domain ratings suggest that certainty is primarily limited by heterogeneity for CET and by imprecision for HYB and RT, and that any ranking information should be viewed as exploratory and considered alongside the magnitude and uncertainty of the relative effects rather than interpreted as definitive.

**Table 1:** CINeMA grades.

Comparison	Number of studies	Within-study bias	Reporting bias	Indirectness	Imprecision	Heterogeneity	Incoherence	Confidence rating
Control:CET	28	Some concerns	Low risk	No concerns	No concerns	Major concerns	No concerns*	Low
Control:CT	12	Some concerns	Low risk	No concerns	Some concerns	Some concerns	No concerns*	Low
Control:HYB	20	Some concerns	Low risk	Some concerns	Major concerns	No concerns	No concerns*	Low
Control:INT	17	Some concerns	Low risk	Some concerns	Some concerns	Some concerns	No concerns*	Low
Control:RT	20	Some concerns	Low risk	No concerns	Major concerns	No concerns	No concerns*	Low

**Abbreviations:** CET, Continuous endurance training; CT, Combined training; HYB, Hybrid or recreational mixed-modality programmes; INT, Interval training; RT, Resistance training

* Incoherence could not be formally assessed using node-splitting or design-by-treatment interaction models because the network contained insufficient closed loops; therefore, quantitative inconsistency testing was not estimable.

### Characteristics of included studies

Table 2 outlines the baseline characteristics of the 66 intervention arms drawn from the 51 RCTs, encompassing 2638 overweight or obese adults (intervention n = 1332; control n = 1306). Twenty-eight arms employed conventional aerobic training (CET) [38,43,47,50,52–54,57,62,64,65,67–70,72,74,75,80,81], 13 used resistance training (RT) [33,40,41,49,56,58,61,62,66,70,72,77,82], 9 implemented hybrid programmes (HYB) [34,35,42,45,47,51,59,60,77], 14 adopted interval training (INT) [32,36–40,43,46,55,58,71,74,76,77] and 8 utilised combined aerobic-resistance training (CT) [35,48,51,63,72,73,78,79]. Twelve trials featured multi-arm designs; all controls maintained usual lifestyle routines [32,35,38,40,47,51,58,62,70,72,74,77].

**Table 2:** Characteristics of the RCTs included in the current systematic review and meta-analysis.

Study	Countries	RCT arms	n (intervention)	n (control)	Intervention characteristics			Main outcomes	Adverse effects
					Type	Length(weeks)	Frequency(times/week)		
Aispuru-Lanche et al.(2024) [32]	Spain	3	28	24	INT	16	2	FMD、CIMT	Not reported
			28	24	INT				
Banks et al.(2024) [33]	United States	2	13	13	RT	9	3	FMD、PWV	Not reported
Barone Gibbs et al.(2024) [34]	United State	2	134	129	HYB	12	7	PWV	Adverse events occurred in both groups; serious adverse events were unrelated to the study, with a few possibly intervention-related musculoskeletal events.
Cox et al.(2024) [35]	Australia	3	11	15	HYB	8	3	FMD、PWV	No adverse events
			15	15	CT		4		
Taha et al.(2023) [36]	Saudi Arabia	2	25	28	INT	12	3	PWV	Not reported
Twerenbold et al.(2023) [37]	Switzerland	2	19	19	INT	8	3	FMD	Not reported
He et al.(2022) [38]	China	4	15	15	CET	8	5	FMD	No adverse events
			8	15	CET	8	3		
			10	15	INT	8	3		
Hovsepian et al.(2021) [39]	Iran	2	14	12	INT	10	4	FMD	One member’s asthma triggered by HIIT
Turri-Silva et al.(2021) [40]	Brazil	3	5	4	INT	12	3	FMD	Safety events were reported: CRT—hypotension, glycemic events and dyspnea; HIIT—one angina episode and one session below anaerobic threshold.
			6	4	RT				
Cahu Rodrigues et al.(2020) [41]	Brazil	2	17	16	RT	12	3	FMD、PWV	No adverse events
Claes et al.(2020) [42]	Belgium	2	60	60	HYB	12	7	FMD、CIMT	Adverse events were similar between groups; no exercise-related adverse events, although four participants withdrew due to serious adverse events.
Ghardashi-Afousi et al.(2020) [43]	Iran	2	30	29	INT	12	3	CIMT	Not reported
Gholami et al.(2020) [44]	Iran	2	16	15	CET	12	3	FMD、CIMT	No adverse events
Hetherington-Rauth et al.(2020) [45]	Portugal	2	14	22	HYB	48	3	PWV、CIMT	Not reported
Ho et al.(2020) [46]	Australia	2	30	30	INT	8	3	PWV	No adverse events
Jo et al.(2020) [47]	South Korea	3	21	13	HYB	12	4	FMD	No adverse events
			13	13	CET				
Jones et al.(2020) [48]	New Zealand	2	26	25	CT	12	2	PWV	No adverse events
Wong et al.(2020) [49]	United States	2	14	14	RT	12	3	PWV	Not reported
Kirkman et al.(2019) [50]	United States	2	15	16	CET	12	3	PWV、FMD	No adverse events
Magalhães et al.(2019) [51]	Portugal	3	16	22	CT	48	3	PWV、CIMT	Not reported
			13	22	HYB				
Rahbar et al.(2018) [52]	Iran	2	13	15	CET	8	3	CIMT	Not reported
Slivovskaja et al.(2018) [53]	Lithuania	2	84	42	CET	8	5	PWV、CIMT	Not reported
Azadpour et al.(2017) [54]	Istanbul	2	12	12	CET	10	3	FMD	Not reported
Bellia et al.(2017) [55]	Italy	2	11	11	INT	12	2	PWV	Not reported
DeVallance et al.(2016) [56]	United States	2	13	16	RT	8	3	CIMT、PWV	Not reported
Robinson et al.(2016) [57]	United States	2	10	9	CET	8	3	FMD	Not reported
Almenning et al.(2015) [58]	Norway	3	8	9	INT	10	3	FMD	Not reported
			8	9	RT				
Figueroa(1) et al.(2015) [59]	United States	2	12	12	HYB	12	3	PWV	Not reported
Figueroa(2) et al.(2014) [60]	United States	2	13	12	HYB	12	3	PWV	Not reported
Franklin et al.(2015) [61]	United States	2	10	8	RT	8	2	FMD	Not reported
Greenwood(1) et al.(2015) [62]	United Kingdom	3	13	20	CET	12	3	PWV	Not reported
					RT				
Greenwood(2) et al.(2015) [63]	United Kingdom	2	8	10	CT	48	3	PWV	Not reported
Oliveira et al.(2015) [64]	Portugal	2	37	41	CET	8	3	PWV	Not reported
Van Craenenbroeck et al.(2015) [65]	Belgium	2	19	21	CET	12	4	FMD	Not reported
Croymans et al.(2014) [66]	United States	2	28	8	RT	12	3	PWV、CIMT	Not reported
Donley et al.(2014) [67]	United States	2	11	11	CET	8	3	PWV、CIMT	Not reported
Headley et al.(2014) [68]	United States	2	25	21	CET	16	3	PWV	Not reported
Pugh et al.(2014) [69]	United Kingdom	2	13	8	CET	16	3	FMD	Not reported
Beck et al.(2013) [70]	United States	3	15	15	RT	8	3	FMD	Not reported
			13	15	CET				
Heydari et al.(2013) [71]	Australia	2	20	18	INT	12	3	PWV	Not reported
Kadoglou et al.(2013) [72]	Greece	4	23	24	RT	24	4	CIMT	Not reported
			21	24	CET				
			22	24	CT				
Dobrosielski et al.(2012) [73]	United States	2	51	63	CT	24	3	PWV	Not reported
Molmen-Hansen et al.(2012) [74]	Norway	3	25	25	INT	12	3	FMD	Not reported
			23	25	CET				
Nualnim et al.(2012) [75]	United States	2	24	19	CET	12	3	PWV、FMD	Not reported
Hermann et al.(2011) [76]	Denmark	2	14	13	INT	8	3	FMD	Not reported
Stensvold et al.(2010) [77]	Norway	4	11	11	INT	12	3	FMD	Not reported
			11	11	RT				
			10	11	HYB				
Loimaala(1) et al.(2009) [78]	Finland	2	24	24	CT	96	4	PWV	Not reported
Loimaala(2) et al.(2003) [79]	Finland	2	24	25	CT	48	4	PWV	Not reported
Braith et al.(2008) [80]	United States	2	9	7	CET	12	3	FMD	Not reported
Bircher et al.(2007) [81]	Germany	2	13	13	CET	12	3	FMD	Not reported
Olson et al.(2006) [82]	United States	2	15	15	RT	48	2	FMD、CIMT	Not reported

**Abbreviations:** RCT arms, number of randomized controlled trial arms; n (intervention), sample size of the intervention group; n (control), sample size of the control group; FMD, flow-mediated dilation; PWV, pulse wave velocity; CIMT, carotid intima-media thickness; CET, continuous endurance training; RT, resistance training; INT, interval training; HYB, hybrid or mixed-modality programmes (e.g., aerobic plus resistance or recreational exercise); CT, combined training (aerobic + resistance).

Adverse-event reporting was inconsistent across included trials and was frequently not reported. Where reported, events were generally mild/manageable, and the trial-level summaries are provided in Table 2 (Adverse effects). Consequently, we did not perform quantitative comparisons of harms across modalities.

### Outcomes

**Fig 2:**
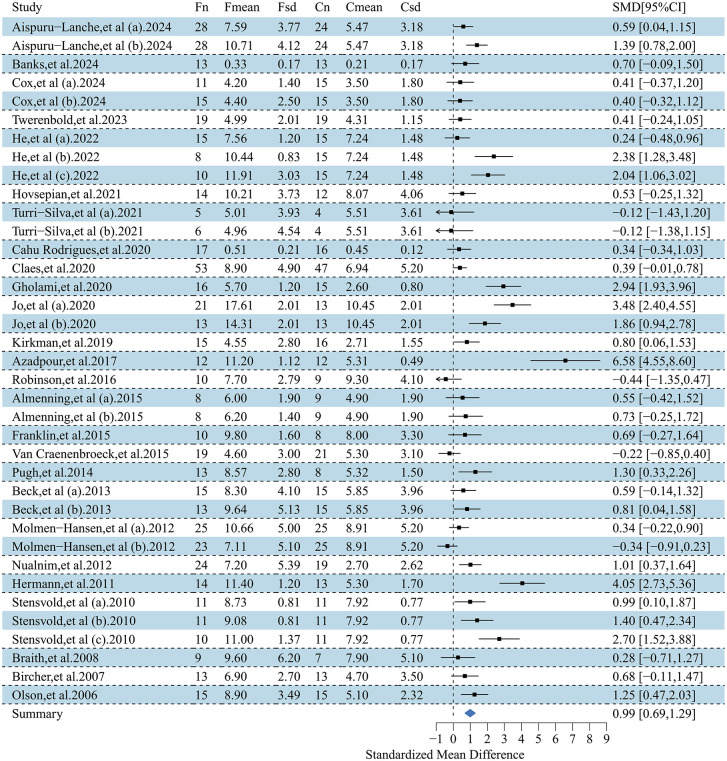
Forest plot for the effect of exercise intervention on flow-mediated dilation (FMD) in overweight or obese adults.

**Fig 3:**
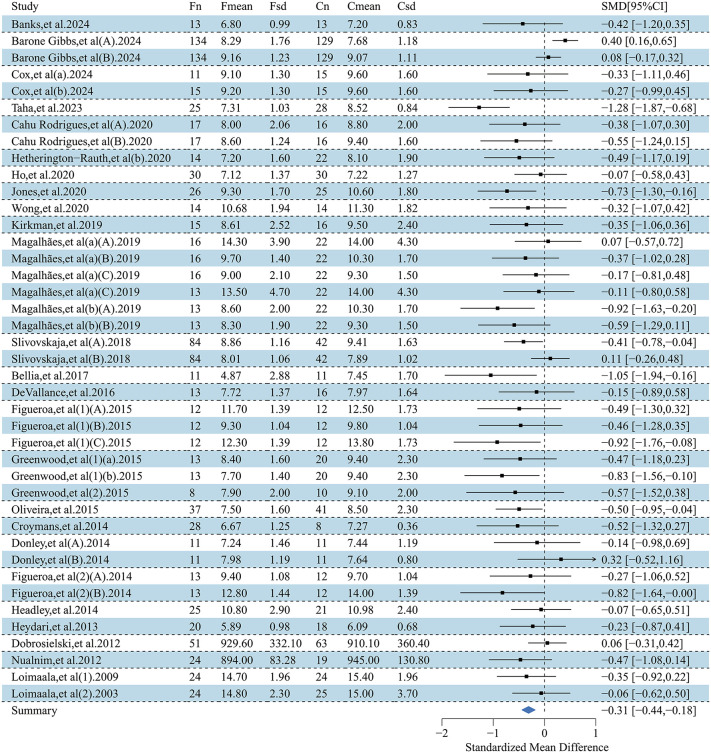
Forest plot for the effect of exercise intervention on pulse wave velocity (PWV) in overweight or obese adults.

**Fig 4:**
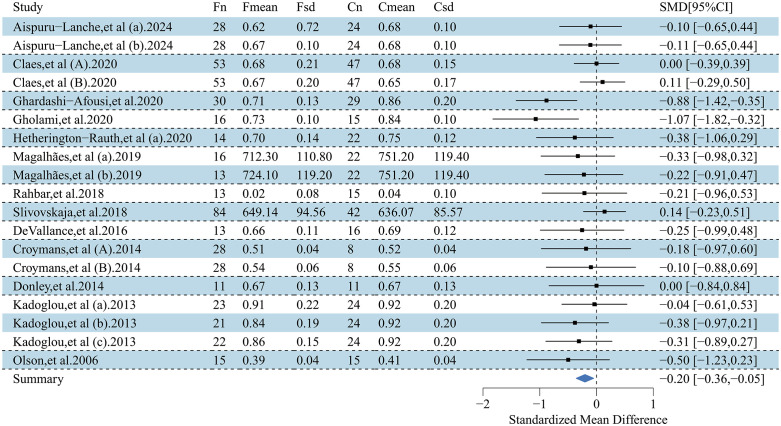
Forest plot for the effect of exercise intervention on intima–media thickness (CIMT) in overweight or obese adults.

This forest plot assessed the effects of exercise interventions on atherosclerosis-related outcomes in adults with obesity. A total of 26 studies (37 effect size) reported a significant improvement in FMD (SMD = 0.99; 95% CI, 0.69–1.29; p < 0.00001), with substantial heterogeneity (I² = 87.1%) [32,33,35,37–42,44,47,50,54,57,58,61,65,69,70,74–77,80–82] (Fig 2). For PWV, 27 studies (41 effect sizes;) demonstrated a significant reduction (SMD = −0.31; 95% CI, −0.44 to −0.18; p < 0.00001), with low-to-moderate heterogeneity (I² = 45.8%) [33–36,41,45,46,48–51,53,55,56,59,60,62–64,66–68,71,73,75,78,79] (Fig 3). To address heterogeneity in PWV acquisition and segment/path definition across trials, we conducted prespecified sensitivity analyses stratified by PWV measurement segment. The pooled effect remained directionally consistent and statistically significant when restricting to central PWV outcomes (k = 22; SMD = −0.185, 95% CI −0.347 to −0.023; I² = 45.3%) and peripheral PWV outcomes (k = 8; SMD = −0.419, 95% CI −0.666 to −0.173; I² = 0%). Results were also robust after excluding studies that reported PWV without a specified segment (k = 30; SMD = −0.241, 95% CI −0.383 to −0.100; I² = 41.1%), indicating that the overall PWV finding was not driven by a specific measurement segment. Additionally, 13 studies (19 effect sizes) showed a significant reduction in CIMT (SMD = −0.20; 95% CI, −0.36 to −0.05; p < 0.05), with low heterogeneity (I² = 21.3%) [32,42–45,51–53,56,66,67,72,82] (Fig 4). Collectively, exercise interventions significantly improve FMD, reduce PWV, and attenuate CIMT in adults with obesity, supporting their role in slowing atherosclerosis progression.

**Fig 5:**
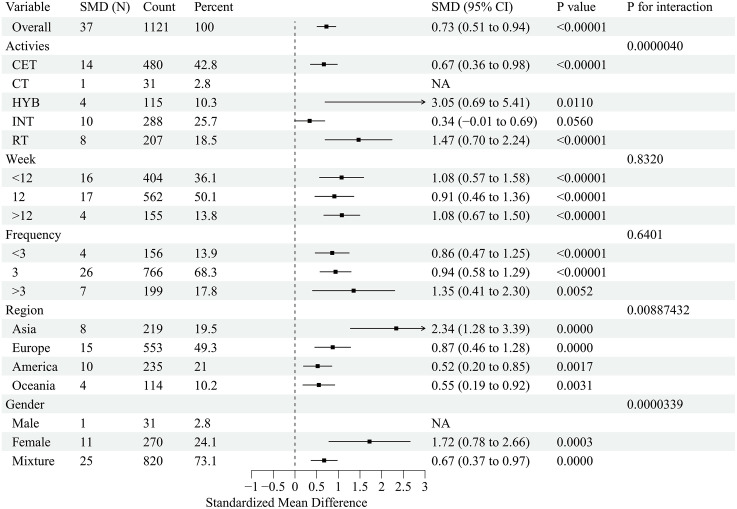
Subgroup analysis of exercise intervention on flow-mediated dilation (FMD) in overweight or obese adults.

**Fig 6:**
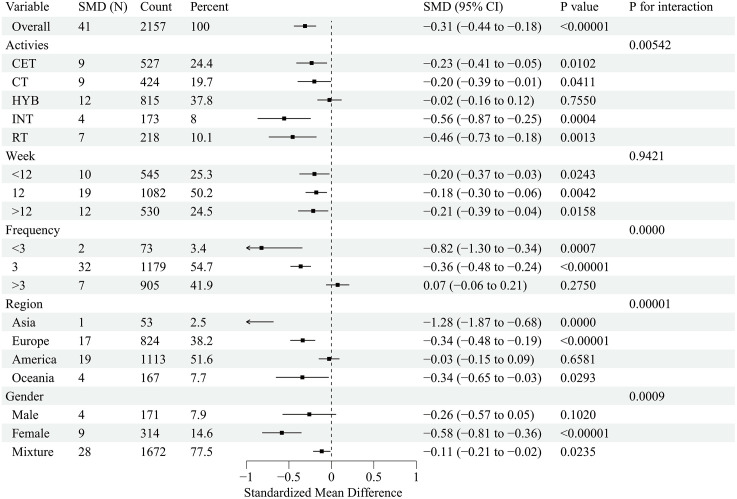
Subgroup analysis of exercise intervention on pulse wave velocity (PWV) in overweight or obese adults.

**Fig 7:**
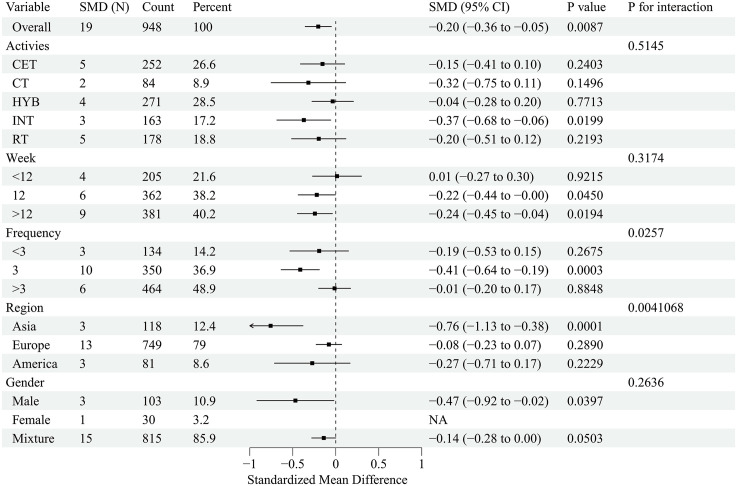
Subgroup analysis of exercise intervention on intima–media thickness (CIMT) in overweight or obese adults.

This subgroup analysis evaluated the effects of exercise interventions on atherosclerosis-related outcomes in adults with obesity. The distribution of female-only and Asia-only trials across modalities and outcome networks is summarised in S5 Table, and these subgroup findings should be interpreted as exploratory given uneven node distributions. FMD improvements were most pronounced in female participants (SMD = 1.72), in Asian studies (SMD = 2.34), with >3 sessions/week (SMD = 1.35) versus <3 sessions/week (SMD = 0.86), and with short-term interventions (<12 weeks; SMD = 1.08) (Fig 5). PWV reductions were greatest in females (SMD = −0.58) and Asian populations (SMD = −1.28), with <3 sessions/week (SMD = −0.82) showing significant effects and interventions >12 weeks being optimal (SMD = −0.21) (Fig 6). For CIMT, the most substantial improvements occurred in Asian populations (SMD = −0.76) and male cohorts (SMD = −0.47), with little difference across frequency and duration subgroups (Fig 7). Collectively, exercise interventions significantly improve FMD, reduce PWV, and attenuateCIMT in adults with obesity. However, optimal strategies appear to differ across vascular indices, suggesting that exercise prescriptions should be tailored to specific targets (FMD, PWV, or CIMT) and individual characteristics (e.g., sex and region) to maximize benefits.

To further explore sources of between-study heterogeneity (I² > 50%), we performed univariate meta-regression analyses to evaluate potential moderators. Each variable—number of intervention arms (RCT arms), adverse events, intervention frequency, intervention duration, gender, and region—was entered separately as a covariate in the regression model. As shown in Table 3, gender (β = −0.978, P = 0.003) and region (β = −0.444, P = 0.022) were significant moderators and were both negatively associated with the intervention effect size. In contrast, RCT arms, adverse events, frequency, and duration were not significant (all P > 0.05).

**Table 3:** Results of meta-regression analyses for Flow-Mediated Dilation (FMD).

Covariate	β (Coefficient)	Std. Err.	t value	P value	95% CI
RCT arms	0.128	0.258	0.50	0.623	–0.395, 0.650
Adverse	0.240	0.207	1.16	0.255	–0.181, 0.660
Frequency	–0.013	0.357	–0.04	0.971	–0.738, 0.712
Duration	–0.053	0.284	–0.19	0.854	–0.630, 0.524
Gender	–0.978	0.312	–3.14	0.003	–1.611, –0.345
Region	–0.444	0.186	–2.39	0.022	–0.821, –0.066

**Abbreviations**: β (Coefficient), regression coefficient; Std. Err., standard error; t value, t statistic; P value, statistical significance; 95% CI, 95% confidence interval; RCT arms, number of trial arms; Adverse, presence/absence of reported adverse events; Frequency, frequency of intervention per week; Duration, total duration of intervention.

**Fig 8:**
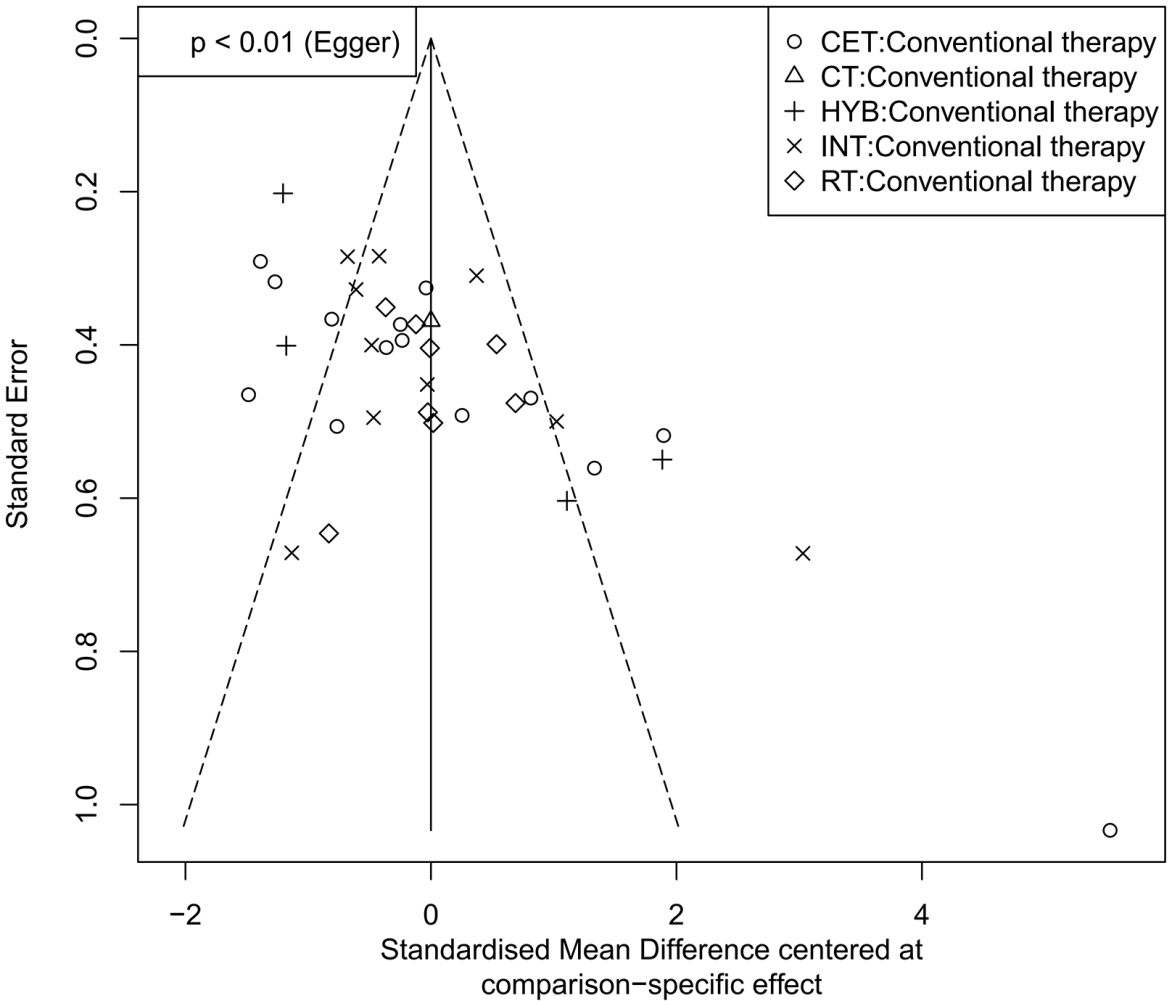
Funnel plot for the assessment of publication bias in studies of exercise intervention on FMD.

**Fig 9:**
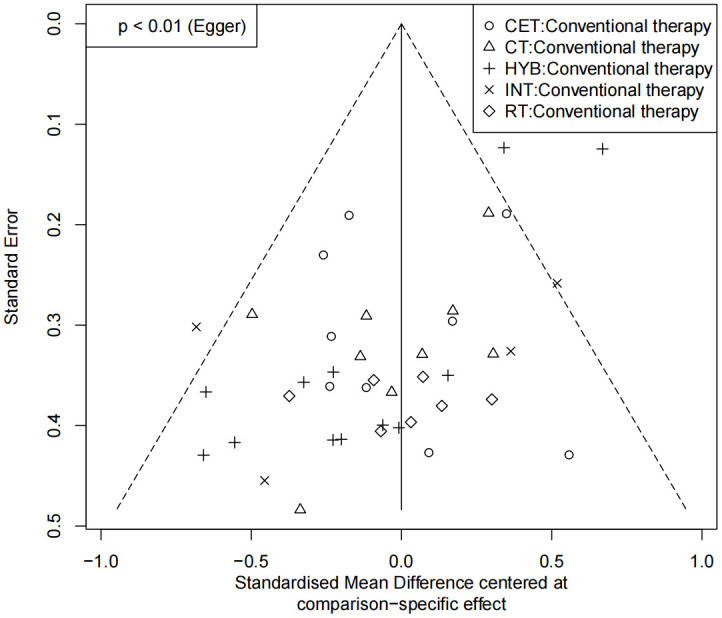
Funnel plot for the assessment of publication bias in studies of exercise intervention on PWV.

**Fig 10:**
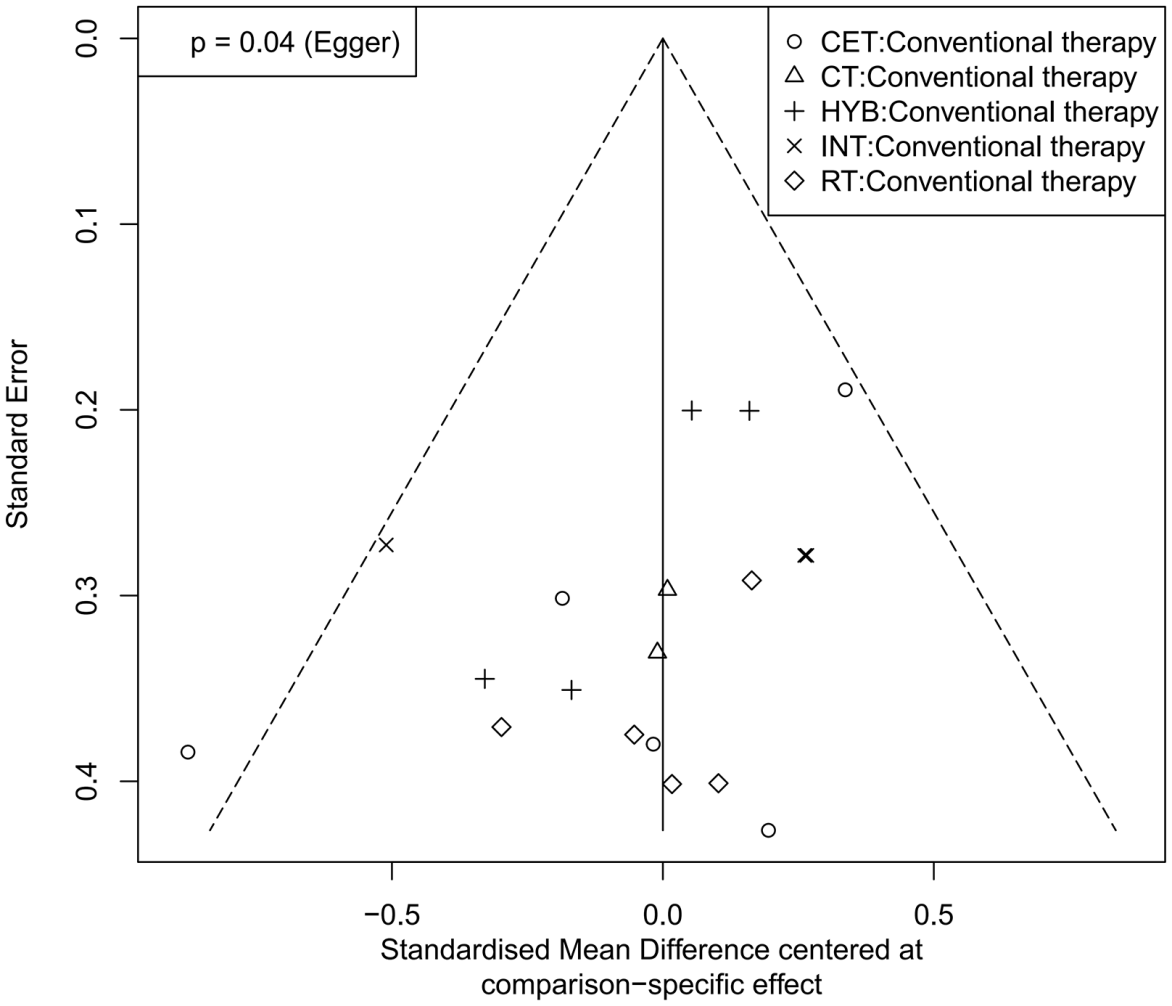
Funnel plot for the assessment of publication bias in studies of exercise intervention on CIMT.

Funnel plots were used to assess potential publication bias and small-study effects for FMD, PWV, and CIMT (Figs 8–10). In the FMD funnel plot, most studies cluster toward the top of the funnel, and as standard errors decrease, data points converge toward the center, forming a generally symmetric inverted funnel shape, suggesting a low likelihood of publication bias. No significant outliers or extreme deviations were observed, further supporting the robustness and reliability of the meta-analysis results.

**Fig 11:**
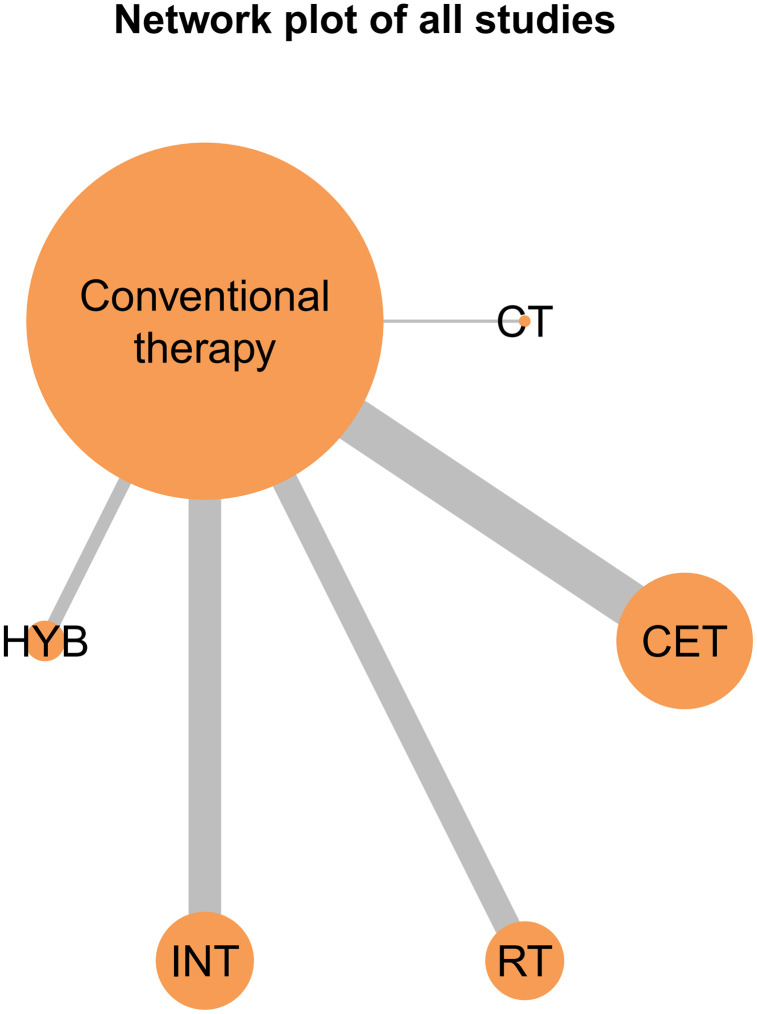
Network evidence plot for the comparative effects of different exercise interventions on FMD.

**Fig 12:**
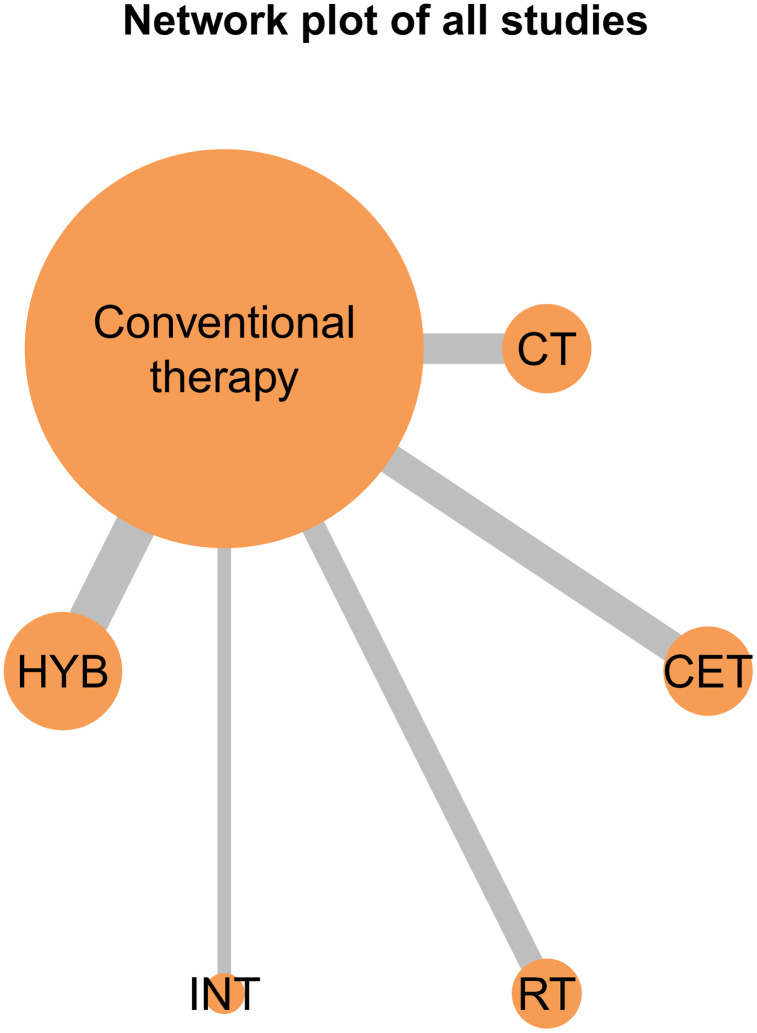
Network evidence plot for the comparative effects of different exercise interventions on PWV.

**Fig 13:**
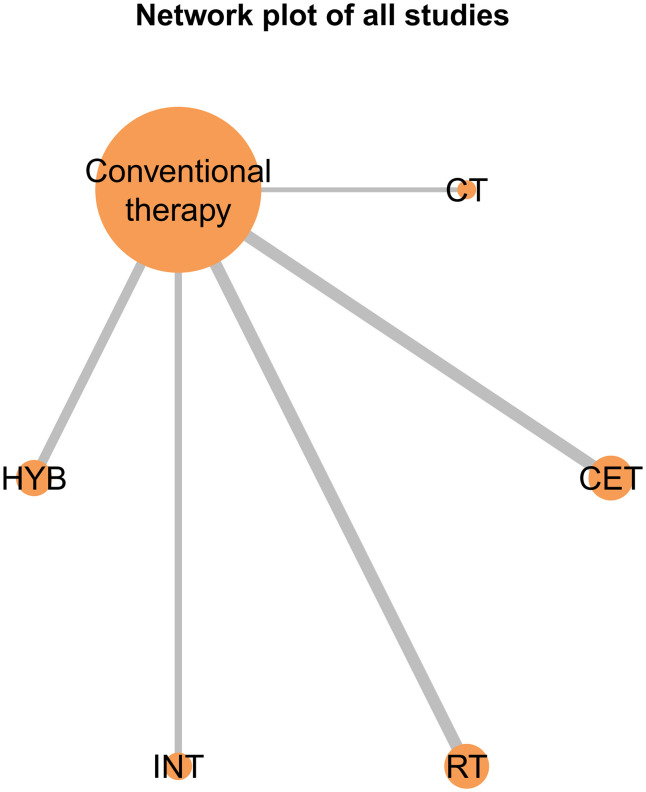
Network evidence plot for the comparative effects of different exercise interventions on CIMT.

The network evidence diagrams for FMD, PWV, and CIMT illustrate the comparative relationships among different exercise interventions. Nodes represent the control group and five intervention types, and connecting lines indicate direct comparisons, with CET being the most frequently studied intervention in the FMD network (Fig 11). These diagrams visualize the frequency of comparisons and provide a clear foundation for the subsequent network meta-analysis. The corresponding network structures for PWV and CIMT are shown in Figs 12 and 13.

**Fig 14:**
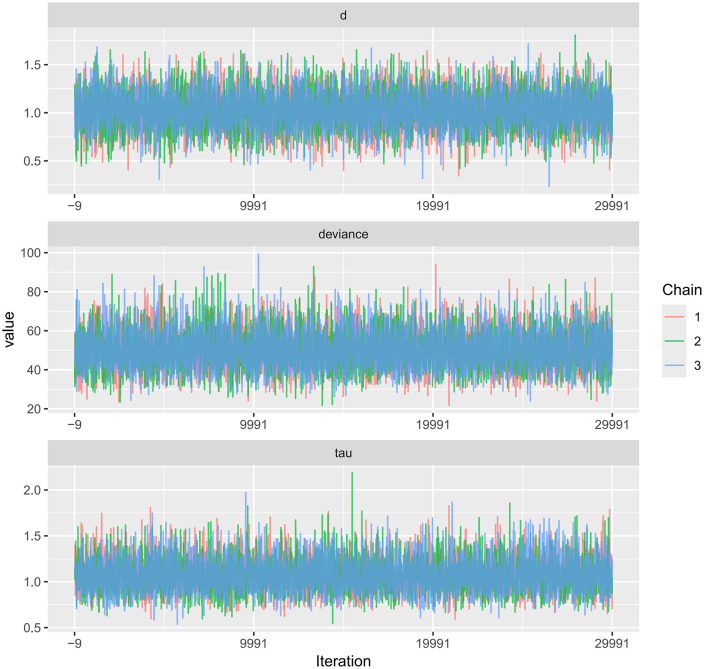
Trace plot for the MCMC convergence diagnostics in Bayesian meta-analysis of FMD.

**Fig 15:**
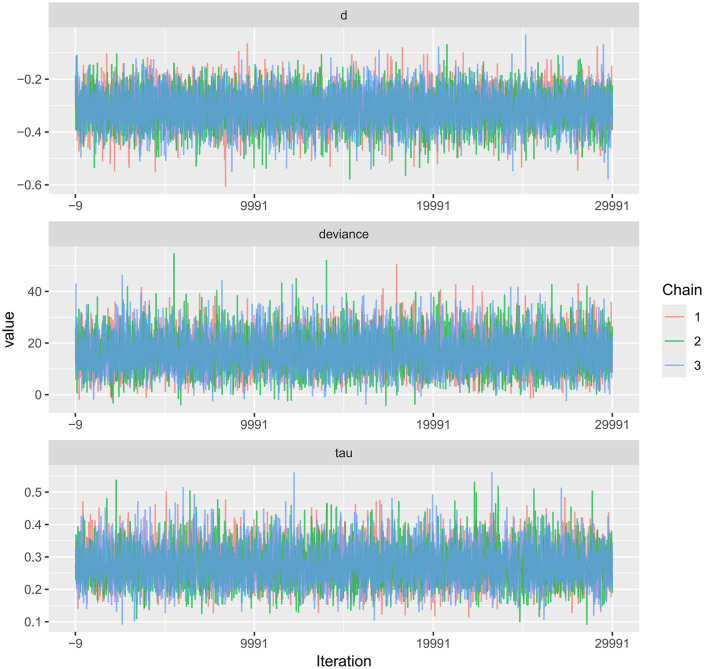
Trace plot for the MCMC convergence diagnostics in Bayesian meta-analysis of PWV.

**Fig 16:**
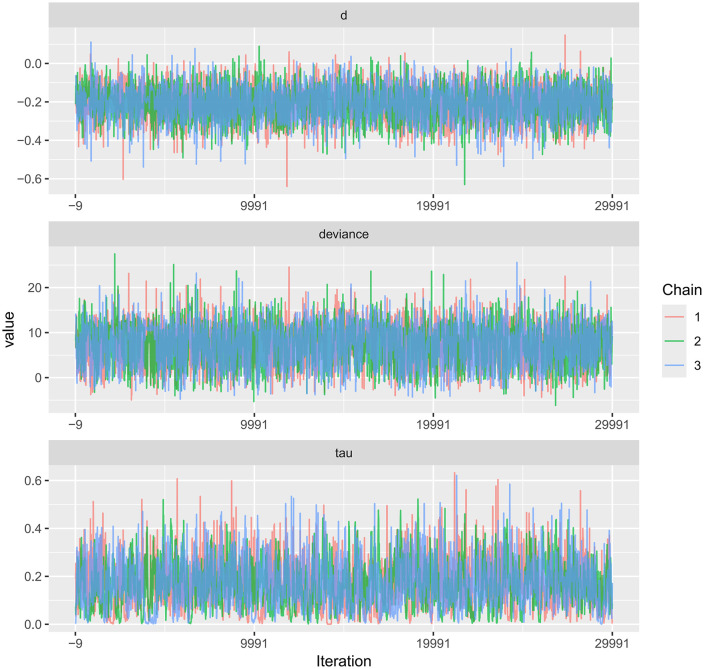
Trace plot for the MCMC convergence diagnostics in Bayesian meta-analysis of CIMT.

Trace plots for PWV and CIMT, as well as their comparative results, are available in the Supplementary Materials.Trace plots were used to visualize the sampling behavior of Markov Chain Monte Carlo (MCMC) iterations and to diagnostically assess convergence of the Bayesian models for FMD, PWV, and CIMT (Figs 14–16). When the number of iterations is insufficient, trace plots typically show pronounced chain fluctuations and poor mixing, indicating inadequate convergence and the need for extended sampling. As shown in the trace plots, once the iteration count reached 7,000, all MCMC chains stabilized after the initial sampling stage and exhibited substantial overlap, becoming visually indistinguishable, which indicates satisfactory mixing and convergence. The chains fluctuated within a consistent range without discernible trends, supporting stable parameter estimates. No abnormal patterns (e.g., periodicity or abrupt jumps) were observed. Collectively, these diagnostics indicate good model convergence and support the reliability of the posterior estimates for all three outcomes.

**Fig 17:**
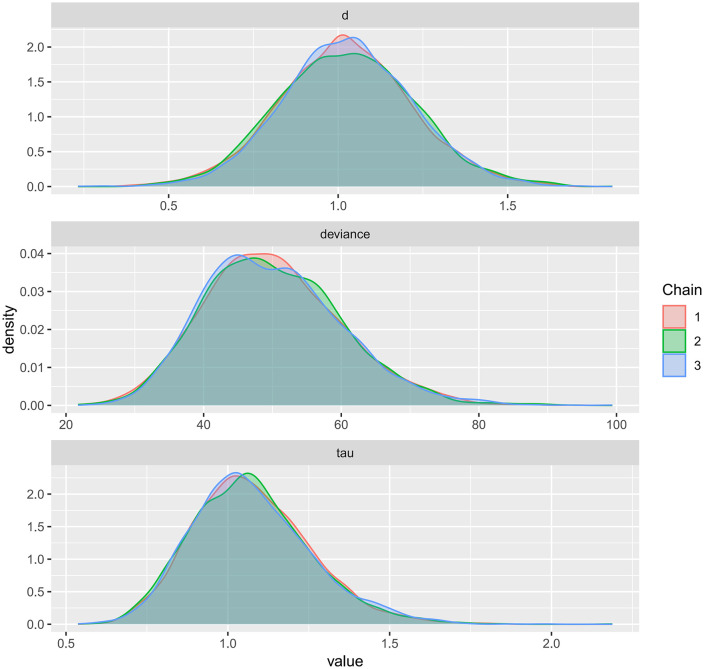
Kernel density plot for the posterior distribution of model parameters in Bayesian meta-analysis of FMD.

**Fig 18:**
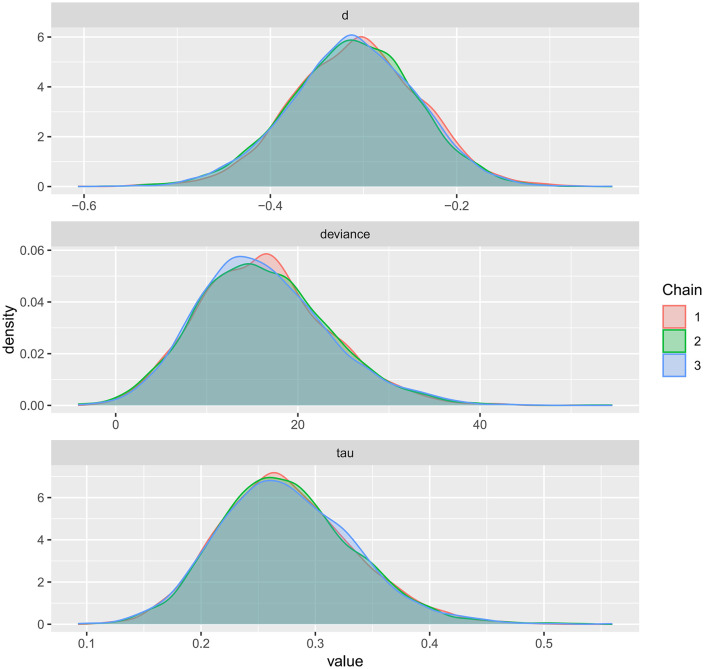
Kernel density plot for the posterior distribution of model parameters in Bayesian meta-analysis of PWV.

**Fig 19:**
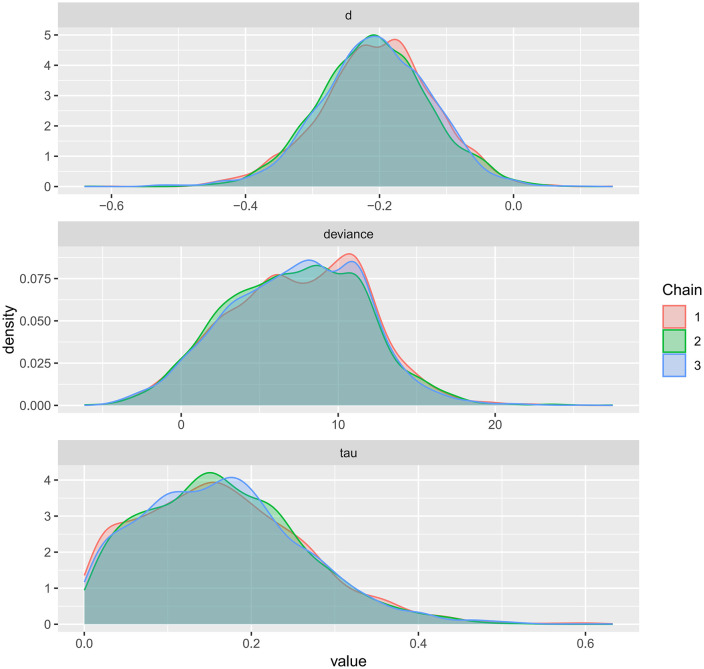
Kernel density plot for the posterior distribution of model parameters in Bayesian meta-analysis of CIMT.

Kernel density plots were used to visualize the posterior distributions of model parameters for FMD, PWV, and CIMT, providing an intuitive representation of uncertainty and central tendency (Figs 17–19). The plots showed smooth, unimodal, and approximately symmetric curves, suggesting stable parameter estimation. The relatively short tails indicated narrow credible intervals, and the distributions exhibited minimal skewness. Compared with the prior distributions, the posteriors were more concentrated, reflecting a strong influence of the observed data on parameter estimation. Overall, these kernel density plots indicate well-formed and reliable posterior estimates, further supporting the robustness of the Bayesian models across all three outcomes.

## Discussion

In this Bayesian network meta-analysis of 51 randomized controlled trials (n = 2,638), we compared five exercise modalities across three complementary vascular indices; FMD reflecting endothelial function; PWV reflecting arterial stiffness and CIMT reflecting structural remodeling. Overall pooled estimates consistently favored exercise, showing a significant improvement in FMD (26 studies; 37 effect sizes; SMD = 0.99, 95% CI 0.69–1.29), a significant reduction in PWV (27 studies; 41 effect sizes; SMD = −0.31, 95% CI −0.44 to −0.18), and a small but statistically significant decrease in CIMT (13 studies; 19 effect sizes; SMD = −0.20, 95% CI −0.36 to −0.05). Notably, the magnitude and variability of effects differed across outcomes: improvements in endothelial function were larger but substantially more heterogeneous (FMD: I² = 87.1%), whereas effects on arterial stiffness and structural indices were smaller but more consistent (PWV: I² = 45.8%; CIMT: I² = 21.3%). This pattern is physiologically plausible and aligns with differences in plasticity and response time across domains: FMD may change over shorter time horizons, PWV reflects elastic/functional properties that may require more sustained exposure, and CIMT—an indicator of structural remodeling—typically changes more slowly and may depend on longer-term metabolic and hemodynamic improvements [83].

A key contribution of this study is the application of Bayesian network meta-analysis to place multiple exercise modalities within a single evidence network, enabling simultaneous comparisons and improving transparency through explicit reporting of network structure and model diagnostics [84]. Using FMD as an example, the network evidence plot demonstrates a fully connected geometry (Fig 11), indicating that even in the absence of direct head-to-head trials between certain modalities, indirect comparisons can be generated via shared comparators. CET occupied a central position with the densest direct evidence versus control, suggesting more stable estimates for CET-related contrasts, whereas sparser comparisons are likely to carry greater uncertainty. We further evaluated model performance using MCMC convergence and posterior diagnostics (Figs 14–19), which supported good chain mixing and stable parameter estimation, thereby strengthening confidence in the network inferences.

Compared with prior work—such as de Oliveira et al. [85], which examined HIIT versus MCT for blood pressure outcomes—the added value of the Bayesian framework in our analysis lies not only in combining direct and indirect evidence across multiple modalities, but also in characterizing uncertainty through posterior distributions and supporting inference with explicit network and convergence diagnostics. Collectively, these features highlight the methodological utility of Bayesian NMA for synthesizing complex intervention networks in exercise science.

Across outcomes, the most pronounced improvement was observed for FMD, and our network comparisons suggested that HYB tended to be the most favorable modality for endothelial function. Ranking uncertainty is illustrated by cumulative rank probability curves and SUCRA summaries (S1 Fig), which show overlap in ranking distributions across modalities for some outcomes; therefore, rankings should be interpreted cautiously and considered alongside the magnitude and uncertainty of the relative effects, particularly where CINeMA ratings indicate low or very low certainty. This outcome-specific pattern (largest effect for FMD compared with PWV and CIMT) supports the interpretation that these indices capture distinct vascular adaptations with different time-scales. As a functional index of endothelium-dependent vasodilation, FMD can respond relatively quickly to repeated hemodynamic stimuli [86–87], which is consistent with the larger and earlier-detectable changes observed for functional outcomes compared with structural remodeling outcomes in our synthesis. HYB may enhance shear stress exposure, which is commonly linked to improved endothelial NO bioavailability through eNOS-related signaling, thereby improving vasodilatory capacity. In this context, the tendency toward greater FMD improvement with HYB in our network is consistent with a shear-stress–NO interpretation, and aligns with prior evidence that moderate-intensity aerobic training improves FMD [88]. However, because eNOS/NO biomarkers were not directly measured in this review, we present this as a biologically plausible explanation rather than a directly verified mediator.Nevertheless, the very high heterogeneity for FMD (I² = 87.1%) indicates substantial between-trial variability, likely attributable to differences in training prescription (intensity, frequency, duration, adherence), baseline cardiometabolic risk, and methodological heterogeneity in FMD assessment (operator expertise, cuff placement, and analytic pipelines). This variability also reinforces that modality rankings for FMD should be interpreted as probabilistic tendencies rather than fixed effects across all contexts.

For arterial stiffness, exercise interventions reduced PWV overall, and network comparisons suggested that interval training (INT) may be more favorable. PWV reflects arterial wall stiffness and is influenced by both structural features and functional tone [89]. By combining aerobic and resistance components, INT may concurrently improve blood pressure regulation, cardiorespiratory fitness, and muscular adaptations, thereby supporting greater gains in arterial compliance [90]. Consistent with the smaller pooled effect size (SMD = −0.31) and low-to-moderate heterogeneity (I² = 45.8%), improvements in PWV appeared more consistent but of modest magnitude. Given imbalances in evidence density across modalities, we frame INT’s apparent advantage as directionally favorable rather than as a definitive top-ranked intervention. This interpretation also aligns with prior reports suggesting that resistance training may exert bidirectional effects on arterial compliance, with excessively high intensities potentially reducing compliance, whereas the inclusion of aerobic components may partially offset such effects.

Regarding structural remodeling, exercise produced a small but significant reduction in CIMT (SMD = −0.20), and network comparisons indicated broadly comparable benefits for CET and RT, with both appearing more favorable than other modalities. As an index of early arterial wall remodeling, CIMT is typically less responsive to short-term interventions, and meaningful changes may require prolonged improvements in hemodynamic and metabolic profiles; thus, a smaller effect size than observed for FMD is expected [91]. Mechanistically, CET may slow CIMT progression through improved hemodynamics, lipid profiles, and insulin sensitivity, whereas RT may act through improved metabolic stress responses and anti-inflammatory effects, including reductions in biomarkers such as C-reactive protein [92]. Prior work has also linked long-term aerobic exercise exposure with slower CIMT progression [93]. By contrast, interval training (INT) and combined training (CT) showed comparatively limited effects on structural outcomes, which may reflect shorter intervention durations, insufficient cumulative training load, or a smaller evidence base, resulting in greater uncertainty [94]. Therefore, current evidence more strongly supports CET and RT for structural endpoints, while the potential structural benefits of INT/CT warrant confirmation in longer and better-powered trials rather than being interpreted as absent.

To explore between-study variability, we conducted subgroup analyses and meta-regression. Subgroup results suggested larger FMD improvements among women and studies conducted in Asian populations, with stronger effects in higher-frequency or shorter-duration interventions; PWV reductions were also greater among women and Asian studies; and CIMT improvements appeared more pronounced in Asian studies and male cohorts. This sex-specific signal aligns with evidence from female-only cardiometabolic cohorts, where structured exercise interventions—including higher-intensity formats—have been linked to favorable cardiometabolic adaptations; however, the populations and endpoints differ from those in our study [95].

Univariate meta-regression indicated that sex and region may be associated with effect-size variability. Importantly, these subgroup and meta-regression findings are based on trial-level aggregated data and may be influenced by differences in trial design, training dose, measurement protocols, and population composition; thus, they should be interpreted as hypothesis-generating rather than as evidence of causal effects of sex or geography.

Finally, although the overall direction of effects favored exercise across all three outcomes, fine-grained comparisons and rankings across modalities require cautious interpretation in light of CINeMA. CINeMA ratings for major control–intervention comparisons were uniformly low, with “some concerns” for within-study bias across contrasts; “major concerns” for heterogeneity in Control:CET (consistent with the high heterogeneity observed for FMD); and “major concerns” for imprecision in Control:HYB and Control:RT, reflecting wide intervals and limited information for certain comparisons. Consequently, while the network suggests that HYB may be more favorable for FMD, INT may be more favorable for PWV, and CET/RT may yield similar effects for CIMT, these apparent advantages should be viewed as probabilistic trends rather than definitive rankings, particularly where evidence is sparse or uncertainty is wide. In practice, modality rankings should be used to support goal-oriented exercise prescription rather than as rigid rules; real-world effectiveness is often driven more by patient preference, feasibility, comorbidity considerations, safety, and long-term adherence than by small between-modality differences.

## Limitations

We must acknowledge several limitations inherent in our study. In addition, harms were not systematically reported in many trials, which limits modality-specific comparisons of safety, particularly for higher-intensity/interval protocols. The included trials exhibited notable heterogeneity in terms of intervention duration, frequency, and intensity. Although we employed a random-effects model and conducted subgroup analyses to mitigate these effects, residual variability may still have introduced bias into our effect estimates. To address this, future research should pursue several key directions: (1) conducting more targeted trials among specific high-risk populations such as older adults and individuals with metabolic syndrome to identify potential subgroup-specific effects; (2) integrating longitudinal multi-indicator tracking to elucidate the dynamic progression of vascular improvements across FMD, PWV, and CIMT domains; and (3) applying machine learning and personalized prediction models to develop individualized exercise recommendations, advancing the paradigm of precision exercise medicine.

## Conclusion

This study evaluated the effects of exercise on vascular health, focusing on FMD, PWV, and CIMT. Exercise interventions showed significant benefits, with HYB improving endothelial function (FMD), INT reducing arterial stiffness (PWV), and both CET and RT showing similar effects on CIMT. The certainty of evidence, assessed using CINeMA, was rated low due to concerns about bias, imprecision, and heterogeneity. Clinically, exercise prescriptions should target the specific outcome: CET for endothelial function, HYB for arterial stiffness, and either CET or RT for CIMT. Long-term adherence and individual feasibility should guide prescription design to ensure sustained benefits.

## Supporting information

S1 Table Detailed search strategy. (DOCX)

S2 Table Exercise intervention classification criteria. (DOCX)

S3 Table Study baseline characteristics and PWV measurement information. (DOCX)

S4 Table Convergence diagnostics for Bayesian models. (DOCX)

S5 Table Distribution of female-only and Asia-only trials across modalities and outcome networks. (DOCX)

S1 Fig Ranking uncertainty of exercise modality effects. (DOCX)

S1 Results Assessment of risk of bias of randomized trials with the Risk of Bias 2. (DOCX)

S1 File PRISMA checklist. (DOCX)

S2 File Effect size computation and imputation of missing standard deviations. (DOCX)

S3 File Bayesian NMA transparency: model specification, priors, software environment, and MCMC settings. (DOCX)
