# Supplementary material for: The effect of exercise intervention on atherosclerosis prevention in overweight or obese adults: A Bayesian network meta-analysis of randomized controlled trials
Source: PLoS One. 2026 Mar 13;21(3):e0344674. doi: 10.1371/journal.pone.0344674 (PMC12987468; doi:10.1371/journal.pone.0344674)
Supplement: S1 Table — (DOCX) [file pone.0344674.s001.docx]

| **Supplementary table S1** Search strategy | | |
| --- | --- | --- |
| **Database** | **strategy** | **Search strategy details** |
| PubMed | 1 | "Overweight"[MeSH Terms] |
|  | 2 | "Obesity"[MeSH Terms] |
|  | 3 | overweight[Title/Abstract] OR obese[Title/Abstract] OR obesity[Title/Abstract] OR "body mass index"[Title/Abstract] OR BMI[Title/Abstract] |
|  | 4 | #1 OR #2 OR #3 |
|  | 5 | "Adult"[MeSH Terms] |
|  | 6 | adult[Title/Abstract] OR adults[Title/Abstract] |
|  | 7 | #5 OR #6 |
|  | 8 | "Exercise"[MeSH Terms] OR "Exercise Therapy"[MeSH Terms] |
|  | 9 | ( (exercise[Title/Abstract]) OR (exercises[Title/Abstract]) OR (training[Title/Abstract]) OR ("physical activity"[Title/Abstract]) OR (aerobic[Title/Abstract]) OR (endurance[Title/Abstract]) OR ("endurance training"[Title/Abstract]) OR ("resistance training"[Title/Abstract]) OR ("resistance exercise"[Title/Abstract]) OR ("strength training"[Title/Abstract]) OR ("interval training"[Title/Abstract]) OR ("high intensity interval"[Title/Abstract]) OR ("high-intensity interval"[Title/Abstract]) OR ("combined training"[Title/Abstract]) OR ("concurrent training"[Title/Abstract]) OR (hybrid[Title/Abstract]) OR ("mixed modality"[Title/Abstract]) OR ("mixed-modality"[Title/Abstract]) OR (multimodal[Title/Abstract]) OR ("multi-modal"[Title/Abstract]) ) |
|  | 10 | #8 OR #9 |
|  | 11 | "Atherosclerosis"[MeSH Terms] |
|  | 12 | ( (atherosclerosis[Title/Abstract]) OR (arteriosclerosis[Title/Abstract]) OR ("arterial stiffness"[Title/Abstract]) OR ("vascular stiffness"[Title/Abstract]) OR ("carotid atherosclerosis"[Title/Abstract]) OR ("vascular health"[Title/Abstract]) OR ("cardiovascular health"[Title/Abstract]) OR ("endothelial function"[Title/Abstract]) OR ("vascular function"[Title/Abstract]) OR ("vascular reactivity"[Title/Abstract]) ) |
|  | 13 | #11 OR #12 |
|  | 14 | "randomized controlled trial"[Publication Type] OR "controlled clinical trial"[Publication Type] |
|  | 15 | randomized[Title/Abstract] OR randomised[Title/Abstract] OR randomly[Title/Abstract] OR trial[Title/Abstract] OR placebo[Title/Abstract] OR "clinical trial"[Title/Abstract] |
|  | 16 | #14 OR #15 |
|  | 17 | #4 AND #7 AND #10 AND #13 AND #16 |
|  | 18 | NOT (animals[MeSH Terms] NOT humans[MeSH Terms]) |
|  | 19 | #17 AND #18 |
| Web of Science | 1 | TS=(overweight OR obese OR obesity OR "body mass index" OR BMI) |
|  | 2 | TS=(adult OR adults) |
|  | 3 | #1 AND #2 |
|  | 4 | TS=(exercise OR exercises OR training OR "physical activity" OR aerobic OR endurance OR "endurance training" OR "resistance training" OR "resistance exercise" OR "strength training" OR "interval training" OR "high intensity interval" OR "high-intensity interval" OR "combined training" OR "concurrent training" OR hybrid OR "mixed modality" OR "mixed-modality" OR multimodal OR "multi-modal") |
|  | 5 | TS=(atherosclerosis OR arteriosclerosis OR "arterial stiffness" OR "vascular stiffness" OR "carotid atherosclerosis" OR "vascular health" OR "cardiovascular health" OR "endothelial function" OR "vascular function" OR "vascular reactivity") |
|  | 6 | TS=(randomized OR randomised OR trial OR placebo OR "controlled trial" OR RCT OR "clinical trial") |
|  | 7 | #3 AND #4 AND #5 AND #6 |
| SPORTDiscus | 1 | (TI(overweight OR obese OR obesity OR "body mass index" OR BMI) OR AB(overweight OR obese OR obesity OR "body mass index" OR BMI) OR KW(overweight OR obese OR obesity OR "body mass index" OR BMI)) |
|  | 2 | (TI(adult OR adults) OR AB(adult OR adults) OR KW(adult OR adults)) |
|  | 3 | #1 AND #2 |
|  | 4 | (TI(exercise OR exercises OR training OR "physical activity" OR aerobic OR endurance OR "endurance training" OR "resistance training" OR "resistance exercise" OR "strength training" OR "interval training" OR HIIT OR "high intensity interval" OR "high-intensity interval" OR "combined training" OR "concurrent training" OR hybrid OR "mixed modality" OR "mixed-modality" OR multimodal OR "multi-modal") OR AB(exercise OR exercises OR training OR "physical activity" OR aerobic OR endurance OR "endurance training" OR "resistance training" OR "resistance exercise" OR "strength training" OR "interval training" OR "high intensity interval" OR "high-intensity interval" OR "combined training" OR "concurrent training" OR hybrid OR "mixed modality" OR "mixed-modality" OR multimodal OR "multi-modal") OR KW(exercise OR exercises OR training OR "physical activity" OR aerobic OR endurance OR "endurance training" OR "resistance training" OR "resistance exercise" OR "strength training" OR "interval training" OR "high intensity interval" OR "high-intensity interval" OR "combined training" OR "concurrent training" OR hybrid OR "mixed modality" OR "mixed-modality" OR multimodal OR "multi-modal")) |
|  | 5 | (TI(atherosclerosis OR arteriosclerosis OR "arterial stiffness" OR "vascular stiffness" OR "carotid atherosclerosis" OR "vascular health" OR "cardiovascular health" OR "endothelial function" OR "vascular function" OR "vascular reactivity") OR AB(atherosclerosis OR arteriosclerosis OR "arterial stiffness" OR "vascular stiffness" OR "carotid atherosclerosis" OR "vascular health" OR "cardiovascular health" OR "endothelial function" OR "vascular function" OR "vascular reactivity") OR KW(atherosclerosis OR arteriosclerosis OR "arterial stiffness" OR "vascular stiffness" OR "carotid atherosclerosis" OR "vascular health" OR "cardiovascular health" OR "endothelial function" OR "vascular function" OR "vascular reactivity")) |
|  | 6 | (TI(randomized OR randomised OR trial OR placebo OR "controlled trial" OR RCT OR "clinical trial") OR AB(randomized OR randomised OR trial OR placebo OR "controlled trial" OR RCT OR "clinical trial") OR KW(randomized OR randomised OR trial OR placebo OR "controlled trial" OR RCT OR "clinical trial")) |
|  | 7 | #3 AND #4 AND #5 AND #6 |
| Cochrane | 1 | (overweight OR obese OR obesity OR "body mass index" OR BMI):ti,ab,kw |
|  | 2 | (adult OR adults):ti,ab,kw |
|  | 3 | #1 AND #2 |
|  | 4 | (exercise OR exercises OR training OR "physical activity" OR aerobic OR endurance OR "endurance training" OR "resistance training" OR "resistance exercise" OR "strength training" OR "interval training" OR "high intensity interval" OR "high-intensity interval" OR "combined training" OR "concurrent training" OR hybrid OR "mixed modality" OR "mixed-modality" OR multimodal OR "multi-modal"):ti,ab,kw |
|  | 5 | (atherosclerosis OR arteriosclerosis OR "arterial stiffness" OR "vascular stiffness" OR "carotid atherosclerosis" OR "vascular health" OR "cardiovascular health" OR "endothelial function" OR "vascular function" OR "vascular reactivity"):ti,ab,kw |
|  | 6 | #3 AND #4 AND #5 |
