# Supplementary material for: The effect of exercise intervention on atherosclerosis prevention in overweight or obese adults: A Bayesian network meta-analysis of randomized controlled trials
Source: PLoS One. 2026 Mar 13;21(3):e0344674. doi: 10.1371/journal.pone.0344674 (PMC12987468; doi:10.1371/journal.pone.0344674)
Supplement: S2 Table — (DOCX) [file pone.0344674.s002.docx]

**Supplementary table S2. Exercise intervention classification criteria.**

| **Type of exercise** | **Definition** |
| --- | --- |
| **CET** | **Frequency:** 3-5x per week  **Intensity:** >45% VO2max or >50% HRR or >65% HRmax  **Time:** 30-60 min per session  **Type:** Any continuous traditional mode of aerobic training only (e.g., walking, running, cycling, rowing, swimming, aerobics, elliptical exercise, and stepping exercise). |
| **INT** | **Frequency:** 2-3x per week  **Intensity:** >65% VO2max or >65% HRR or >75% HRmax  **Gross exercise time:** 20-30 min per session  **Type:** Any intermittent traditional mode of interval training, including single-component MIIT and HIIT (e.g., walking, running, cycling, rowing, swimming, elliptical exercise, and stepping exercise). |
| **RT** | **Frequency:** 2-3x per week  **Intensity:** >=50% 1RM  **Gross exercise time:** 30-60 min per session  **Type:** Any mode of resistance training, including circuit-based programs (e.g., free weights, weight machines, and resistance bands). |
| **CT** | A combination of CET and RT. |
| **HYB** | **Frequency:** 2-3x per week  **Intensity:** >65% VO2max or >65% HRR or >75% HRmax  **Gross exercise time:** 30-45 min per session  **Type:** Any intermittent multicomponent exercise mode engaging both the cardiovascular and the musculoskeletal systems throughout a single exercise session using both muscle-strengthening and dynamic cardiovascular exercises (e.g., small-sided games prescribed as structured training, high-intensity functional training, integrated neuromuscular training, cardio-resistance training, and multimodal training). |
| **CON** | No exercise. |

**Abbreviations:** CET, continuous endurance training; CON, control; CT, combined training; HIIT, high-intensity interval training; HRmax, maximum heart rate; HRR, heart rate reserve; HYB, hybrid-type training; INT, interval training; MIIT, moderate-intensity interval training; RM, repetition maximum; RT, resistance training; VO2max, maximal oxygen uptake.
