# Supplementary material for: The effect of exercise intervention on atherosclerosis prevention in overweight or obese adults: A Bayesian network meta-analysis of randomized controlled trials
Source: PLoS One. 2026 Mar 13;21(3):e0344674. doi: 10.1371/journal.pone.0344674 (PMC12987468; doi:10.1371/journal.pone.0344674)
Supplement: S3 Table — (DOCX) [file pone.0344674.s003.docx]

**Supplementary table S3.** Study baseline characteristics and PWV measurement information

| **Study** | **Outcome(s)** | **Arm (node)** | **N** | **Female (%)** | **Age (mean±SD)** | **Baseline BMI (mean±SD)** | **Baseline FMD (mean±SD)** | **Baseline PWV (mean±SD)** | **Baseline CIMT (mean±SD)** | **PWV measure(s)** | **PWV domain** |
| --- | --- | --- | --- | --- | --- | --- | --- | --- | --- | --- | --- |
| Aispuru-Lanche,et al (a) | FMD、CIMT | INT | 28 | 14.3 | 59.0±9.6 | 30.5±6.8 | NR | NR | NR | NR | NR |
| Aispuru-Lanche,et al (a) | FMD、CIMT | Control | 24 | 20.8 | 57.0±7.2 | 28.1±5.4 | NR | NR | NR | NR | NR |
| Aispuru-Lanche,et al (b) | FMD、CIMT | INT | 28 | 17.9 | 58.9±8.0 | 30.7±4.9 | NR | NR | NR | NR | NR |
| Aispuru-Lanche,et al (b) | FMD、CIMT | Control | 24 | 20.8 | 57.0±7.2 | 28.1±5.4 | NR | NR | NR | NR | NR |
| Banks,et al | FMD、PWV | RT | 13 | 61.5 | 52 (6) | 29.9 (4.2) | 0.24 (0.1) | 7.0 (0.5) | NR | cfPWV | central |
| Banks,et al | FMD、PWV | Control | 13 | 61.5 | 55 (6) | 28.0 (4.3) | 0.20 (0.1) | 7.3 (0.6) | NR | cfPWV | central |
| Barone Gibbs,et al（A） | PWV | HYB | 134 | 59.6 | 47.2±11.4 | 30.6±5.9 | NR | 8.2±1.7 | NR | cfPWV; crPWV | central |
| Barone Gibbs,et al（A） | PWV | Control | 129 | 60.7 | 43.2±11.5 | 31.2±7.2 | NR | 7.7±1.2 | NR | cfPWV; crPWV | central |
| Barone Gibbs,et al（B） | PWV | HYB | 134 | 59.6 | 47.2±11.4 | 30.6±5.9 | NR | 9.1±1.3 | NR | cfPWV; crPWV | central |
| Barone Gibbs,et al（B） | PWV | Control | 129 | 60.7 | 43.2±11.5 | 31.2±7.2 | NR | 9.0±1.1 | NR | cfPWV; crPWV | central |
| Cox,et al (a) | FMD、PWV | HYB | 11 | 39.1 | 59.0±8.8 | 32.6±5.1 | 3.6±1.7 | 8.7±1.6 | NR | cfPWV | central |
| Cox,et al (a) | FMD、PWV | Control | 15 | 39.1 | 59.4±10.2 | 33.9±6.4 | 3.8±2.1 | 9.6±1.8 | NR | cfPWV | central |
| Cox,et al (b) | FMD、PWV | CT | 15 | 39.1 | 60.1±7.3 | 34.0±6.6 | 3.9±2.0 | 9.2±1.3 | NR | cfPWV | central |
| Cox,et al (b) | FMD、PWV | Control | 15 | 39.1 | 59.4±10.2 | 33.9±6.4 | 3.8±2.1 | 9.6±1.8 | NR | cfPWV | central |
| Taha,et al | PWV | INT | 25 | 100 | 48.32 ± 4.48 | 33.90 ± 2.58 | NR | 8.45 ± 0.99 | NR | PWV | unspecified |
| Taha,et al | PWV | Control | 28 | 100 | 48.92 ± 3.60 | 34.90 ± 1.53 | NR | 8.37 ± 0.91 | NR | PWV | unspecified |
| Twerenbold,et al | FMD | INT | 19 | 31.6 | 56 ± 6 | 25.7 ± 3.1 | 4.90 ± 2.35 | NR | NR | NR | NR |
| Twerenbold,et al | FMD | Control | 19 | 42.1 | 59 ± 7 | 25.2 ± 2.9 | 4.35 ± 1.31 | NR | NR | NR | NR |
| He,et al (a) | FMD | CET | 15 | 100 | 57.6 ± 12.39 | 23.5 ± 1.94 | NR | NR | NR | NR | NR |
| He,et al (a) | FMD | Control | 15 | 100 | 58.33 ± 11.85 | 24.9 ± 2.71 | NR | NR | NR | NR | NR |
| He,et al (b) | FMD | CET | 8 | 100 | 54.3 ± 18.10 | 25.6 ± 2.26 | NR | NR | NR | NR | NR |
| He,et al (b) | FMD | Control | 15 | 100 | 58.33 ± 11.85 | 24.9 ± 2.71 | NR | NR | NR | NR | NR |
| He,et al (c) | FMD | INT | 10 | 100 | 55.8 ± 17.71 | 23.2 ± 1.90 | NR | NR | NR | NR | NR |
| He,et al (c) | FMD | Control | 15 | 100 | 58.33 ± 11.85 | 24.9 ± 2.71 | NR | NR | NR | NR | NR |
| Hovsepian,et al | FMD | INT | 14 | 100 | 53.9 ± 3.39 | 28.2 ± 3.45 | 7.63 ± 1.11 | NR | NR | NR | NR |
| Hovsepian,et al | FMD | Control | 12 | 100 | 53.2 ± 3.07 | 27.4 ± 3.51 | 7.06 ± 1.42 | NR | NR | NR | NR |
| Turri-Silva,et al (a) | FMD | INT | 5 | 37.5 | 60.9 ± 9.7 | 29.4 ± 5.2 | 5.37±2.59 | NR | NR | NR | NR |
| Turri-Silva,et al (a) | FMD | Control | 4 | 12.5 | 56.0 ± 9.7 | 28.6 ± 4.5 | 4.62±2.60 | NR | NR | NR | NR |
| Turri-Silva,et al (b) | FMD | RT | 6 | 33.3 | 55.0 ± 10.9 | 32.9 ± 6.5 | 4.54±2.65 | NR | NR | NR | NR |
| Turri-Silva,et al (b) | FMD | Control | 4 | 12.5 | 56.0 ± 9.7 | 28.6 ± 4.5 | 4.62±2.60 | NR | NR | NR | NR |
| Cahu Rodrigues,et al | FMD | RT | 17 | NR | 61 ± 8.25 | 32.0 ± 4.12 | 0.47 ± 0.12 | NR | NR | cPWV; pPWV | mixed (central+peripheral) |
| Cahu Rodrigues,et al | FMD | Control | 16 | NR | 59 ± 8.00 | 29.8 ± 6.80 | 0.48 ± 0.16 | NR | NR | cPWV; pPWV | mixed (central+peripheral) |
| Cahu Rodrigues,et al（A） | PWV | RT | 17 | NR | 61 ± 8.25 | 32.0 ± 4.12 | NR | 9.1 ± 2.06 | NR | cPWV; pPWV | mixed (central+peripheral) |
| Cahu Rodrigues,et al（A） | PWV | Control | 16 | NR | 59 ± 8.00 | 29.8 ± 6.80 | NR | 8.7 ± 2.00 | NR | cPWV; pPWV | mixed (central+peripheral) |
| Cahu Rodrigues,et al（B） | PWV | RT | 17 | NR | 61 ± 8.25 | 32.0 ± 4.12 | NR | 8.4 ± 1.24 | NR | cPWV; pPWV | mixed (central+peripheral) |
| Cahu Rodrigues,et al（B） | PWV | Control | 16 | NR | 59 ± 8.00 | 29.8 ± 6.80 | NR | 9.4 ± 1.60 | NR | cPWV; pPWV | mixed (central+peripheral) |
| Claes,et al | FMD | HYB | 53 | 18.3 | 61.7±14.5 | 27.4±3.50 | 8.10±7.40 | NR | NR | NR | NR |
| Claes,et al | FMD | Control | 47 | 18.3 | 59.6±13.2 | 28.2±5.30 | 8.00±6.40 | NR | NR | NR | NR |
| Claes,et al（A） | CIMT | HYB | 53 | 18.3 | 61.7±14.5 | 27.4±3.50 | 8.10±7.40 | NR | 0.72±0.15 | NR | NR |
| Claes,et al（A） | CIMT | Control | 47 | 18.3 | 59.6±13.2 | 28.2±5.30 | 8.00±6.40 | NR | 0.71±0.16 | NR | NR |
| Claes,et al（B） | CIMT | HYB | 53 | 18.3 | 61.7±14.5 | 27.4±3.50 | 8.10±7.40 | NR | 0.68±0.17 | NR | NR |
| Claes,et al（B） | CIMT | Control | 47 | 18.3 | 59.6±13.2 | 28.2±5.30 | 8.00±6.40 | NR | 0.65±0.16 | NR | NR |
| Ghardashi-Afousi,et al | CIMT | INT | 30 | 50.0 | 55.10 ± 6.07 | 30.47 ± 1.32 | NR | NR | 0.83 ± 0.17 | NR | NR |
| Ghardashi-Afousi,et al | CIMT | Control | 29 | 44.8 | 54.10 ± 5.68 | 29.48 ± 1.25 | NR | NR | 0.84 ± 0.20 | NR | NR |
| Gholami,et al | FMD、CIMT | CET | 16 | 0.0 | 53.4 ± 9.1 | 28.2 ± 2.5 | 3.2 ± 1.1 | NR | 0.78 ± 0.1 | NR | NR |
| Gholami,et al | FMD、CIMT | Control | 15 | 0.0 | 52.2 ± 8.5 | 28.7 ± 1.8 | 2.8 ± 0.9 | NR | 0.82 ± 0.1 | NR | NR |
| Hetherington-Rauth,et al（a） | PWV、CIMT | HYB | 14 | 42.9 | 56.6 ± 4.9 | 30.4 ± 6.1 | NR | 13.2 ± 3.6 | 0.70 ± 0.13 | Aortic PWV | central |
| Hetherington-Rauth,et al（a） | PWV、CIMT | Control | 22 | 50.0 | 60.8 ± 7.5 | 31.7 ± 4.7 | NR | 13.1 ± 4.8 | 0.71 ± 0.13 | Aortic PWV | central |
| Ho,et al | PWV | INT | 30 | 100 | 53.9 ± 3.39 | 28.2 ± 3.45 | NR | 7.63 ± 1.11 | NR | baPWV | peripheral |
| Ho,et al | PWV | Control | 30 | 100 | 53.2 ± 3.07 | 27.4 ± 3.51 | NR | 7.06 ± 1.42 | NR | baPWV | peripheral |
| Jo,et al (a) | FMD | HYB | 21 | 100 | 61.8 ± 10.1 | 27.7 ± 3.0 | NR | NR | NR | NR | NR |
| Jo,et al (a) | FMD | Control | 13 | 100 | 62.5 ± 13.9 | 27.3 ± 4.6 | NR | NR | NR | NR | NR |
| Jo,et al (b) | FMD | CET | 13 | 100 | 57.3 ± 8.4 | 27.0 ± 3.0 | NR | NR | NR | NR | NR |
| Jo,et al (b) | FMD | Control | 13 | 100 | 62.5 ± 13.9 | 27.3 ± 4.6 | NR | NR | NR | NR | NR |
| Jones,et al | PWV | CT | 26 | 100 | 55.8 ± 7.2 | 27.8 ± 5.5 | NR | 9.6 ± 2.0 | NR | PWV | unspecified |
| Jones,et al | PWV | Control | 25 | 100 | 55.9 ± 7.1 | 27.5 ± 4.8 | NR | 10.2 ± 1.4 | NR | PWV | unspecified |
| Wong,et al | PWV | RT | 14 | 100 | 22.0 ± 3.74 | 34.3 ± 2.99 | NR | NR | NR | baPWV | peripheral |
| Wong,et al | PWV | Control | 14 | 100 | 23.0 ± 3.74 | 34.4 ± 3.74 | NR | NR | NR | baPWV | peripheral |
| Kirkman,et al | PWV、FMD | CET | 15 | 31.3 | 55 ± 13 | 30 ± 2 | 2.61 ± 1.56 | 8.73 ± 2.12 | NR | PWV | unspecified |
| Kirkman,et al | PWV | Control | 16 | 26.7 | 62 ± 9 | 34 ± 6 | 3.48 ± 2.44 | 9.76 ± 1.90 | NR | PWV | unspecified |
| Magalhães,et al（a） | CIMT | CT | 16 | 53.6 | 59.7 ± 6.5 | 31.1 ± 5.0 | NR | NR | 723.1 ± 142.5 | cdPWV; cfPWV; crPWV | central |
| Magalhães,et al（a） | CIMT | Control | 22 | 48.1 | 59.0 ± 8.1 | 30.7 ± 5.0 | NR | NR | 716.5 ± 120.6 | cdPWV; cfPWV; crPWV | central |
| Magalhães,et al（a）（A） | PWV | CT | 16 | 53.6 | 59.7 ± 6.5 | 31.1 ± 5.0 | NR | 13.0 ± 3.3 | NR | cdPWV; cfPWV; crPWV | central |
| Magalhães,et al（a）（A） | PWV | Control | 22 | 48.1 | 59.0 ± 8.1 | 30.7 ± 5.0 | NR | 12.9 ± 4.4 | NR | cdPWV; cfPWV; crPWV | central |
| Magalhães,et al（a）（B） | PWV | CT | 16 | 53.6 | 59.7 ± 6.5 | 31.1 ± 5.0 | NR | 10.1 ± 1.8 | NR | cdPWV; cfPWV; crPWV | central |
| Magalhães,et al（a）（B） | PWV | Control | 22 | 48.1 | 59.0 ± 8.1 | 30.7 ± 5.0 | NR | 9.2 ± 2.0 | NR | cdPWV; cfPWV; crPWV | central |
| Magalhães,et al（a）（C） | PWV | CT | 16 | 53.6 | 59.7 ± 6.5 | 31.1 ± 5.0 | NR | 9.6 ± 1.6 | NR | cdPWV; cfPWV; crPWV | central |
| Magalhães,et al（a）（C） | PWV | Control | 22 | 48.1 | 59.0 ± 8.1 | 30.7 ± 5.0 | NR | 9.2 ± 1.9 | NR | cdPWV; cfPWV; crPWV | central |
| Magalhães,et al（b） | CIMT | HYB | 13 | 40 | 56.7 ± 8.3 | 30.1 ± 5.7 | NR | NR | 713.2 ± 175.8 | cdPWV; cfPWV; crPWV | central |
| Magalhães,et al（b） | CIMT | Control | 22 | 48.1 | 59.0 ± 8.1 | 30.7 ± 5.0 | NR | NR | 716.5 ± 120.6 | cdPWV; cfPWV; crPWV | central |
| Magalhães,et al（b）（A） | PWV | HYB | 13 | 40 | 56.7 ± 8.3 | 30.1 ± 5.7 | NR | 13.2 ± 3.7 | NR | cdPWV; cfPWV; crPWV | central |
| Magalhães,et al（b）（A） | PWV | Control | 22 | 48.1 | 59.0 ± 8.1 | 30.7 ± 5.0 | NR | 12.9 ± 4.4 | NR | cdPWV; cfPWV; crPWV | central |
| Magalhães,et al（b）（B） | PWV | HYB | 13 | 40 | 56.7 ± 8.3 | 30.1 ± 5.7 | NR | 10.2 ± 2.7 | NR | cdPWV; cfPWV; crPWV | central |
| Magalhães,et al（b）（B） | PWV | Control | 22 | 48.1 | 59.0 ± 8.1 | 30.7 ± 5.0 | NR | 9.2 ± 2.0 | NR | cdPWV; cfPWV; crPWV | central |
| Magalhães,et al（b）（C） | PWV | HYB | 13 | 40 | 56.7 ± 8.3 | 30.1 ± 5.7 | NR | 9.5 ± 1.4 | NR | cdPWV; cfPWV; crPWV | central |
| Magalhães,et al（b）（C） | PWV | Control | 22 | 48.1 | 59.0 ± 8.1 | 30.7 ± 5.0 | NR | 9.2 ± 1.9 | NR | cdPWV; cfPWV; crPWV | central |
| Rahbar,et al | CIMT | CET | 13 | NR | 48.31 ± 5.02 | 48.60 ± 4.80 | NR | NR | 0.09 ± 0.02 | NR | NR |
| Rahbar,et al | CIMT | Control | 15 | NR | 48.60 ± 4.80 | 26.93 ± 2.42 | NR | NR | 0.10 ± 0.03 | NR | NR |
| Slivovskaja,et al | CIMT | CET | 84 | 59.5 | 53.89 ± 6.43 | 30.89 ± 3.97 | NR | NR | 648.9 ± 105.18 | cfPWV; crPWV | central |
| Slivovskaja,et al | CIMT | Control | 42 | 42.9 | 52.01 ± 7.67 | 30.97 ± 3.51 | NR | NR | 628.24 ± 97.05 | cfPWV; crPWV | central |
| Slivovskaja,et al（A） | PWV | CET | 84 | 59.5 | 53.89 ± 6.43 | 30.89 ± 3.97 | NR | 9.15 ± 1.20 | NR | cfPWV; crPWV | central |
| Slivovskaja,et al（A） | PWV | Control | 42 | 42.9 | 52.01 ± 7.67 | 30.97 ± 3.51 | NR | 9.04 ± 1.06 | NR | cfPWV; crPWV | central |
| Slivovskaja,et al（B） | PWV | CET | 84 | 59.5 | 53.89 ± 6.43 | 30.89 ± 3.97 | NR | 8.47 ± 1.40 | NR | cfPWV; crPWV | central |
| Slivovskaja,et al（B） | PWV | Control | 42 | 42.9 | 52.01 ± 7.67 | 30.97 ± 3.51 | NR | 8.01 ± 1.13 | NR | cfPWV; crPWV | central |
| Azadpour,et al | FMD | CET | 12 | 100 | 57.58 ± 4.29 | 32.15 ± 1.78 | 6.02 ± 0.71 | NR | NR | NR | NR |
| Azadpour,et al | FMD | Control | 12 | 100 | 56.58 ± 4.17 | 31.29 ± 1.40 | 5.78 ± 0.52 | NR | NR | NR | NR |
| Bellia,et al | PWV | INT | 11 | 18.2 | 58.8 ± 7.9 | 27.7 ± 2.8 | NR | 7.73 ± 1.70 | NR | PWV | unspecified |
| Bellia,et al | PWV | Control | 11 | 36.4 | 56.3 ± 6.4 | 29.9 ± 3.4 | NR | 7.40 ± 0.93 | NR | PWV | unspecified |
| DeVallance,et al | PWV、CIMT | RT | 13 | 69 | 51 ± 10.82 | 32 ± 3.61 | NR | NR | 0.68 ± 0.072 | cfPWV | central |
| DeVallance,et al | PWV、CIMT | Control | 16 | 73 | 51 ± 16.00 | 33 ± 8.00 | NR | NR | 0.71 ± 0.200 | cfPWV | central |
| Robinson,et al | FMD | CET | 10 | 70.0 | 34 ± 8 | 32 ± 5 | 8.6 ± 4.8 | NR | NR | NR | NR |
| Robinson,et al | FMD | Control | 9 | 77.8 | 28 ± 5 | 33 ± 6 | 9.3 ± 4.2 | NR | NR | NR | NR |
| Almenning,et al (a) | FMD | INT | 8 | 100 | NR | 26.1 ± 6.5 | 4.0 ± 1.2 | NR | NR | NR | NR |
| Almenning,et al (a) | FMD | Control | 9 | 100 | NR | 27.4 ± 6.9 | 6.2 ± 1.9 | NR | NR | NR | NR |
| Almenning,et al (b) | FMD | RT | 8 | 100 | NR | 27.4 ± 6.9 | 5.7 ± 2.2 | NR | NR | NR | NR |
| Almenning,et al (b) | FMD | Control | 9 | 100 | NR | 27.4 ± 6.9 | 6.2 ± 1.9 | NR | NR | NR | NR |
| Figueroa,et al(1)（A） | PWV | HYB | 12 | 100 | 58 ± 3.46 | 34.6 ± 3.12 | NR | 12.2 ± 1.73 | NR | Aortic PWV; baPWV; legPWV | mixed (central+peripheral) |
| Figueroa,et al(1)（A） | PWV | Control | 12 | 100 | 58 ± 3.46 | 32.2 ± 7.27 | NR | 12.1 ± 2.08 | NR | Aortic PWV; baPWV; legPWV | mixed (central+peripheral) |
| Figueroa,et al(1)（B） | PWV | HYB | 12 | 100 | 58 ± 3.46 | 34.6 ± 3.12 | NR | 10.2 ± 1.04 | NR | Aortic PWV; baPWV; legPWV | mixed (central+peripheral) |
| Figueroa,et al(1)（B） | PWV | Control | 12 | 100 | 58 ± 3.46 | 32.2 ± 7.27 | NR | 9.9 ± 1.04 | NR | Aortic PWV; baPWV; legPWV | mixed (central+peripheral) |
| Figueroa,et al(1)（C） | PWV | HYB | 12 | 100 | 58 ± 3.46 | 34.6 ± 3.12 | NR | 13.6 ± 1.73 | NR | Aortic PWV; baPWV; legPWV | mixed (central+peripheral) |
| Figueroa,et al(1)（C） | PWV | Control | 12 | 100 | 58 ± 3.46 | 32.2 ± 7.27 | NR | 13.8 ± 1.73 | NR | Aortic PWV; baPWV; legPWV | mixed (central+peripheral) |
| Franklin,et al | FMD | RT | 10 | 100 | 30.3 ± 5.4 | 34.2 ± 3.0 | 9.5 ± 1.6 | NR | NR | NR | NR |
| Franklin,et al | FMD | Control | 8 | 100 | 30.8 ± 9.0 | 32.2 ± 6.9 | 8.4 ± 3.5 | NR | NR | NR | NR |
| Greenwood,et al(1)（a） | PWV | CET | 13 | 23.1 | 53.9 ± 10.7 | 26.6 ± 4.7 | NR | 9.0 ± 1.4 | NR | PWV | unspecified |
| Greenwood,et al(1)（a） | PWV | Control | 20 | 50.0 | 49.5 ± 10.6 | 27.3 ± 3.6 | NR | 8.9 ± 2.3 | NR | PWV | unspecified |
| Greenwood,et al(1)（b） | PWV | RT | 13 | 46.2 | 54.6 ± 10.6 | 28.2 ± 3.6 | NR | 9.1 ± 1.8 | NR | PWV | unspecified |
| Greenwood,et al(1)（b） | PWV | Control | 20 | 50.0 | 49.5 ± 10.6 | 27.3 ± 3.6 | NR | 8.9 ± 2.3 | NR | PWV | unspecified |
| Greenwood,et al(2) | PWV | CT | 8 | 25 | 53.8 ± 13.5 | 27.40 ± 3.52 | NR | 9.5 ± 2.2 | NR | PWV | unspecified |
| Greenwood,et al(2) | PWV | Control | 10 | 10 | 53.3 ± 12.9 | 28.44 ± 4.24 | NR | 8.1 ± 2.4 | NR | PWV | unspecified |
| Oliveira,et al | PWV | CET | 37 | 13.6 | 55.0 ± 10.7 | 26.57 ± 3.41 | NR | 8.0 ± 2.2 | NR | cfPWV | central |
| Oliveira,et al | PWV | Control | 41 | 19.0 | 58.5 ± 10.7 | 27.07 ± 2.74 | NR | 8.4 ± 2.1 | NR | cfPWV | central |
| Van Craenenbroeck,et al | FMD | CET | 19 | 42 | 51.5 ± 11.8 | 28.3 ± 6.2 | 4.0 ± 1.9 | NR | NR | NR | NR |
| Van Craenenbroeck,et al | FMD | Control | 21 | 48 | 54.7 ± 14.1 | 28.3 ± 5.8 | 5.2 ± 3.4 | NR | NR | NR | NR |
| Croymans,et al | PWV | RT | 28 | 0.0 | 21.5 ± 2.3 | 31.1 ± 2.3 | NR | 6.77 ± 0.94 | NR | cfPWV | central |
| Croymans,et al | PWV | Control | 8 | 0.0 | 21.9 ± 1.8 | 33.2 ± 3.1 | NR | 6.93 ± 0.80 | NR | cfPWV | central |
| Croymans,et al（A） | CIMT | RT | 28 | 0.0 | 21.5 ± 2.3 | 31.1 ± 2.3 | NR | NR | 0.523 ± 0.039 | cfPWV | central |
| Croymans,et al（A） | CIMT | Control | 8 | 0.0 | 21.9 ± 1.8 | 33.2 ± 3.1 | NR | NR | 0.493 ± 0.054 | cfPWV | central |
| Croymans,et al（B） | CIMT | RT | 28 | 0.0 | 21.5 ± 2.3 | 31.1 ± 2.3 | NR | NR | 0.523 ± 0.039 | cfPWV | central |
| Croymans,et al（B） | CIMT | Control | 8 | 0.0 | 21.9 ± 1.8 | 33.2 ± 3.1 | NR | NR | 0.520 ± 0.054 | cfPWV | central |
| Donley,et al | CIMT | CET | 11 | 73 | 46 ± 13.27 | 38 ± 6.63 | NR | NR | 0.70 ± 0.133 | cfPWV; crPWV | central |
| Donley,et al | CIMT | Control | 11 | 64 | 44 ± 9.95 | 34 ± 6.63 | NR | NR | 0.69 ± 0.199 | cfPWV; crPWV | central |
| Donley,et al（A） | PWV | CET | 11 | 73 | 46 ± 13.27 | 38 ± 6.63 | NR | 7.93 ± 1.92 | NR | cfPWV; crPWV | central |
| Donley,et al（A） | PWV | Control | 11 | 64 | 44 ± 9.95 | 34 ± 6.63 | NR | 7.45 ± 1.46 | NR | cfPWV; crPWV | central |
| Donley,et al（B） | PWV | CET | 11 | 73 | 46 ± 13.27 | 38 ± 6.63 | NR | 7.93 ± 1.33 | NR | cfPWV; crPWV | central |
| Donley,et al（B） | PWV | Control | 11 | 64 | 44 ± 9.95 | 34 ± 6.63 | NR | 7.76 ± 0.73 | NR | cfPWV; crPWV | central |
| Figueroa,et al(2)（A） | PWV | HYB | 13 | 100 | 55.5 ± 2.52 | 33.6 ± 4.69 | NR | 10.2 ± 1.08 | NR | Aortic PWV; baPWV; legPWV | mixed (central+peripheral) |
| Figueroa,et al(2)（A） | PWV | Control | 12 | 100 | 56.4 ± 3.46 | 36.0 ± 3.12 | NR | 9.8 ± 1.04 | NR | Aortic PWV; baPWV; legPWV | mixed (central+peripheral) |
| Figueroa,et al(2)（B） | PWV | HYB | 13 | 100 | 55.5 ± 2.52 | 33.6 ± 4.69 | NR | 14.1 ± 2.16 | NR | Aortic PWV; baPWV; legPWV | mixed (central+peripheral) |
| Figueroa,et al(2)（B） | PWV | Control | 12 | 100 | 56.4 ± 3.46 | 36.0 ± 3.12 | NR | 14.1 ± 1.04 | NR | Aortic PWV; baPWV; legPWV | mixed (central+peripheral) |
| Headley,et al | PWV | CET | 25 | 0.0 | 24.9±4.3 | 28.4±2.4 | NR | NR | NR | Aortic PWV | central |
| Headley,et al | PWV | Control | 21 | 0.0 | 24.9±4.3 | 29.0±3.9 | NR | NR | NR | Aortic PWV | central |
| Pugh,et al | FMD | CET | 13 | 46.1 | 50.0 ± 10.82 | 30.0 ± 2.48 | 4.79 ± 2.23 | NR | NR | NR | NR |
| Pugh,et al | FMD | Control | 8 | 50 | 47.0 ± 14.14 | 30.0 ± 4.78 | 5.94 ± 1.93 | NR | NR | NR | NR |
| Beck,et al (a) | FMD | RT | 15 | 26.7 | 21.1 ± 2.5 | 27.4 ± 5.1 | 6.17 ± 3.07 | NR | NR | NR | NR |
| Beck,et al (a) | FMD | Control | 15 | 33.3 | 21.6 ± 2.9 | 27.0 ± 4.3 | 6.20 ± 3.69 | NR | NR | NR | NR |
| Beck,et al (b) | FMD | CET | 13 | 30.8 | 20.1 ± 1.1 | 28.7 ± 5.5 | 5.92 ± 3.80 | NR | NR | NR | NR |
| Beck,et al (b) | FMD | Control | 15 | 33.3 | 21.6 ± 2.9 | 27.0 ± 4.3 | 6.20 ± 3.69 | NR | NR | NR | NR |
| Heydari,et al | PWV | INT | 20 | 0.0 | 24.9±4.3 | 28.4±2.4 | NR | NR | NR | PWV | unspecified |
| Heydari,et al | PWV | Control | 18 | 0.0 | 24.9±4.3 | 29.0±3.9 | NR | NR | NR | PWV | unspecified |
| Kadoglou,et al（a） | CIMT | RT | 23 | 69.6 | 56.1 ± 5.3 | 32.89 ± 3.26 | NR | NR | 0.806 ± 0.237 | NR | NR |
| Kadoglou,et al（a） | CIMT | Control | 24 | 70.8 | 57.9 ± 7.2 | 32.1 ± 2.95 | NR | NR | 0.786 ± 0.218 | NR | NR |
| Kadoglou,et al（b） | CIMT | CET | 21 | 71.4 | 58.3 ± 5.4 | 31.55 ± 3.11 | NR | NR | 0.822 ± 0.195 | NR | NR |
| Kadoglou,et al（b） | CIMT | Control | 24 | 70.8 | 57.9 ± 7.2 | 32.1 ± 2.95 | NR | NR | 0.786 ± 0.218 | NR | NR |
| Kadoglou,et al（c） | CIMT | CT | 22 | 77.3 | 57.9 ± 6.5 | 31.91 ± 2.93 | NR | NR | 0.795 ± 0.169 | NR | NR |
| Kadoglou,et al（c） | CIMT | Control | 24 | 70.8 | 57.9 ± 7.2 | 32.1 ± 2.95 | NR | NR | 0.786 ± 0.218 | NR | NR |
| Dobrosielski,et al | PWV | CT | 51 | 41.0 | 57 ± 6 | 33.0 ± 5.02 | NR | 922.1 ± 335.50 | NR | PWV | unspecified |
| Dobrosielski,et al | PWV | Control | 63 | 43.0 | 56 ± 6 | 33.6 ± 4.18 | NR | 909.4 ± 358.09 | NR | PWV | unspecified |
| Molmen-Hansen,et al (a) | FMD | INT | 25 | 48.4 | 52.5±7.4 | 26.8±4.1 | 6.49±3.71 | NR | NR | NR | NR |
| Molmen-Hansen,et al (a) | FMD | Control | 25 | 41.4 | 51.3±9.2 | 28.8±3.7 | 8.01±4.62 | NR | NR | NR | NR |
| Molmen-Hansen,et al (b) | FMD | CET | 23 | 42.9 | 53.6±6.5 | 27.9±3.2 | 6.50±5.01 | NR | NR | NR | NR |
| Molmen-Hansen,et al (b) | FMD | Control | 25 | 41.4 | 51.3±9.2 | 28.8±3.7 | 8.01±4.62 | NR | NR | NR | NR |
| Nualnim,et al | FMD、PWV | CET | 24 | 70.8 | 58 ± 9.80 | 29 ± 4.90 | 3.3 ± 4.41 | 914 ± 97.98 | NR | Femoral–anklePWV | peripheral |
| Nualnim,et al | FMD、PWV | Control | 19 | 79 | 61 ± 8.72 | 32 ± 4.36 | 4.8 ± 3.92 | 940 ± 74.10 | NR | Femoral–anklePWV | peripheral |
| Hermann,et al | FMD | INT | 14 | 14.3 | 53 ± 11 | 26.3 ± 3.4 | 8.3 ± 1.3 | NR | NR | NR | NR |
| Hermann,et al | FMD | Control | 13 | 23.1 | 47 ± 18 | 26.1 ± 6.1 | 5.6 ± 1.0 | NR | NR | NR | NR |
| Stensvold,et al (a) | FMD | INT | 11 | NR | 49.9 ± 10.1 | 31.3 ± 4.3 | NR | NR | NR | NR | NR |
| Stensvold,et al (a) | FMD | Control | 11 | NR | 47.3 ± 10.2 | 31.9 ± 4.1 | NR | NR | NR | NR | NR |
| Stensvold,et al (b) | FMD | RT | 11 | NR | 50.9 ± 7.6 | 32.2 ± 4.2 | NR | NR | NR | NR | NR |
| Stensvold,et al (b) | FMD | Control | 11 | NR | 47.3 ± 10.2 | 31.9 ± 4.1 | NR | NR | NR | NR | NR |
| Stensvold,et al (c) | FMD | HYB | 10 | NR | 52.9 ± 10.4 | 30.3 ± 3.5 | NR | NR | NR | NR | NR |
| Stensvold,et al (c) | FMD | Control | 11 | NR | 47.3 ± 10.2 | 31.9 ± 4.1 | NR | NR | NR | NR | NR |
| Loimaala,et al(1) | PWV | CT | 24 | 0.0 | 53.6 ± 6.2 | 29.3 ± 3.7 | NR | 14.1 ± 2.45 | NR | PWV | unspecified |
| Loimaala,et al(1) | PWV | Control | 24 | 0.0 | 54.0 ± 5.0 | 29.8 ± 3.6 | NR | 14.1 ± 2.45 | NR | PWV | unspecified |
| Braith,et al | FMD | CET | 9 | 22 | 54.4 ± 13.1 | 25.5 ± 3.6 | 10.1 ± 6.1 | NR | NR | NR | NR |
| Braith,et al | FMD | Control | 7 | 14 | 54.3 ± 9.5 | 28.5 ± 1.4 | 10.5 ± 2.8 | NR | NR | NR | NR |
| Bircher,et al | FMD | CET | 13 | NR | 50.1 ± 7.8 | 33.0 ± 6.1 | 4.3 ± 3.5 | NR | NR | NR | NR |
| Bircher,et al | FMD | Control | 13 | NR | 51.8 ± 7.5 | 34.8 ± 3.6 | 4.9 ± 2.9 | NR | NR | NR | NR |
| Olson,et al | FMD、CIMT | RT | 15 | 100 | 38.0 ± 3.87 | 27.5 ± 3.49 | 6.3 ± 2.71 | NR | 0.50 ± 0.15 | NR | NR |
| Olson,et al | FMD、CIMT | Control | 15 | 100 | 38.0 ± 7.75 | 27.6 ± 2.71 | 6.5 ± 2.32 | NR | 0.46 ± 0.12 | NR | NR |
| Loimaala,et al(2) | PWV | CT | 24 | 0.0 | 53.6 ± 6.2 | 29.3 ± 3.8 | NR | 14.2 ± 2.6 | NR | PWV | unspecified |
| Loimaala,et al(2) | PWV | Control | 25 | 0.0 | 54.0 ± 5.0 | 29.8 ± 3.7 | NR | 13.8 ± 2.7 | NR | PWV | unspecified |

**Table note:** Values are presented as mean ± SD unless otherwise specified. Female (%) is calculated as the proportion of women in each group. Baseline vascular outcomes (FMD, PWV, CIMT) refer to pre-intervention measurements. PWV measurement type/segment was extracted as reported in each trial; when PWV was reported without a specified segment, it was classified as “unspecified PWV”. PWV domain was categorized as central (cfPWV, aortic PWV, and other central derivatives) or peripheral (baPWV, leg PWV, femoral–ankle PWV). Abbreviations: FMD, flow-mediated dilation; PWV, pulse wave velocity; CIMT, carotid intima–media thickness; cfPWV, carotid–femoral pulse wave velocity; baPWV, brachial–ankle pulse wave velocity; SD, standard deviation.
