# Supplementary material for: The effect of exercise intervention on atherosclerosis prevention in overweight or obese adults: A Bayesian network meta-analysis of randomized controlled trials
Source: PLoS One. 2026 Mar 13;21(3):e0344674. doi: 10.1371/journal.pone.0344674 (PMC12987468; doi:10.1371/journal.pone.0344674)
Supplement: S4 Table — (DOCX) [file pone.0344674.s004.docx]

**Supplementary table S4. Convergence diagnostics for Bayesian models**

This table summarizes quantitative convergence diagnostics across all monitored parameters (relative effects, heterogeneity τ, and meta-regression coefficients where applicable) for each fitted model.

| **Outcome / model** | **Parameters monitored** | **Max R-hat (across parameters)** | **Min bulk ESS (across parameters)** | **Min tail ESS (across parameters)** | **Chains** | **Iter / chain** | **Warmup / chain** | **Thinning** | **Sampler controls (adapt_delta, max_treedepth)** |
| --- | --- | --- | --- | --- | --- | --- | --- | --- | --- |
| BNMA model (brms_result) | relative effects; τ; (meta-reg coeffs if used) | 1.000179 | 21773.42 | 29882.34 | 3 | 50000 | 20000 | 1 | adapt_delta = 0.95; max_treedepth = default |

**Table note.**

R-hat values and effective sample sizes (ESS) were computed from posterior draws using the posterior package. For each model, we report the maximum R-hat and the minimum bulk and tail ESS across all monitored parameters (excluding non-inferential sampler outputs such as lp__ and other internal variables). Values close to 1.00 for R-hat and sufficiently large ESS indicate satisfactory mixing and convergence.

Software environment (as reported in S2 Appendix): R 4.4.2; brms 2.22.0; rstan 2.32.6; Stan 2.32.2.
