# Supplementary material for: The effect of exercise intervention on atherosclerosis prevention in overweight or obese adults: A Bayesian network meta-analysis of randomized controlled trials
Source: PLoS One. 2026 Mar 13;21(3):e0344674. doi: 10.1371/journal.pone.0344674 (PMC12987468; doi:10.1371/journal.pone.0344674)
Supplement: S5 Table — (DOCX) [file pone.0344674.s005.docx]

**Supplementary Table S5. Distribution of female-only and Asia-only trials by modality and outcome network**

Counts (k) represent the number of trials within each outcome network that exclusively enrolled women (female-only) or were conducted in Asia (Asia-only), stratified by exercise modality. These distributions were used to contextualize subgroup findings by sex and region.

**Panel A. Female-only trials (k) by modality**

| **Outcome network** | **CET** | **RT** | **INT** | **HYB** | **CT** | **Total** |
| --- | --- | --- | --- | --- | --- | --- |
| FMD | 3 | 3 | 3 | 1 | 0 | 10 |
| PWV | 0 | 1 | 2 | 1 | 1 | 5 |
| CIMT | 0 | 1 | 0 | 0 | 0 | 1 |

**Panel B. Asia-only trials (k) by modality**

| **Outcome network** | **CET** | **RT** | **INT** | **HYB** | **CT** | **Total** |
| --- | --- | --- | --- | --- | --- | --- |
| FMD | 4 | 0 | 2 | 1 | 0 | 7 |
| PWV | 0 | 0 | 1 | 0 | 0 | 1 |
| CIMT | 2 | 0 | 1 | 0 | 0 | 3 |

**Table note.**

Female-only trials were defined as trials enrolling 100% women. Asia-only trials were defined as trials conducted in Asian countries/regions. Modality categories follow the prespecified intervention grouping used in the main analyses. Counts may differ across outcome networks because not all trials reported all outcomes.

**Abbreviations:**

CET, continuous endurance training; RT, resistance training; INT, interval training; HYB, hybrid/combined modalities as defined in the review; CT, combined training; FMD, flow-mediated dilation; PWV, pulse wave velocity; CIMT, carotid intima–media thickness.
