# Supplementary material for: The effect of exercise intervention on atherosclerosis prevention in overweight or obese adults: A Bayesian network meta-analysis of randomized controlled trials
Source: PLoS One. 2026 Mar 13;21(3):e0344674. doi: 10.1371/journal.pone.0344674 (PMC12987468; doi:10.1371/journal.pone.0344674)
Supplement: S1 Fig — (DOCX) [file pone.0344674.s006.docx]

**S1 Fig. Cumulative rank probability curves (SUCRA) for ranking uncertainty across exercise modalities for FMD, PWV, and CIMT.**

**
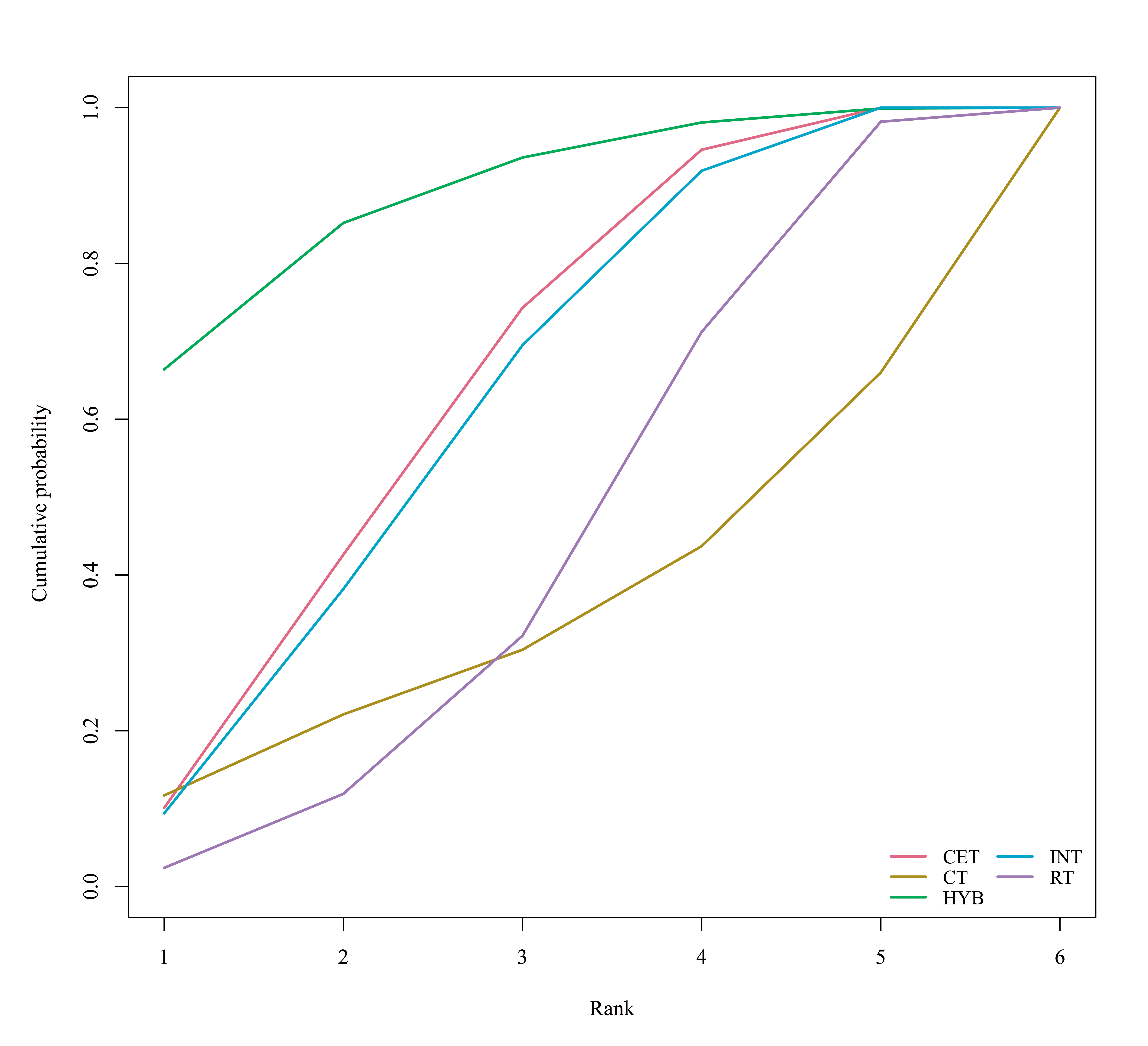
Fig S3.** Cumulative rank probability curves (SUCRA) for ranking uncertainty across exercise modalities for FMD.

**
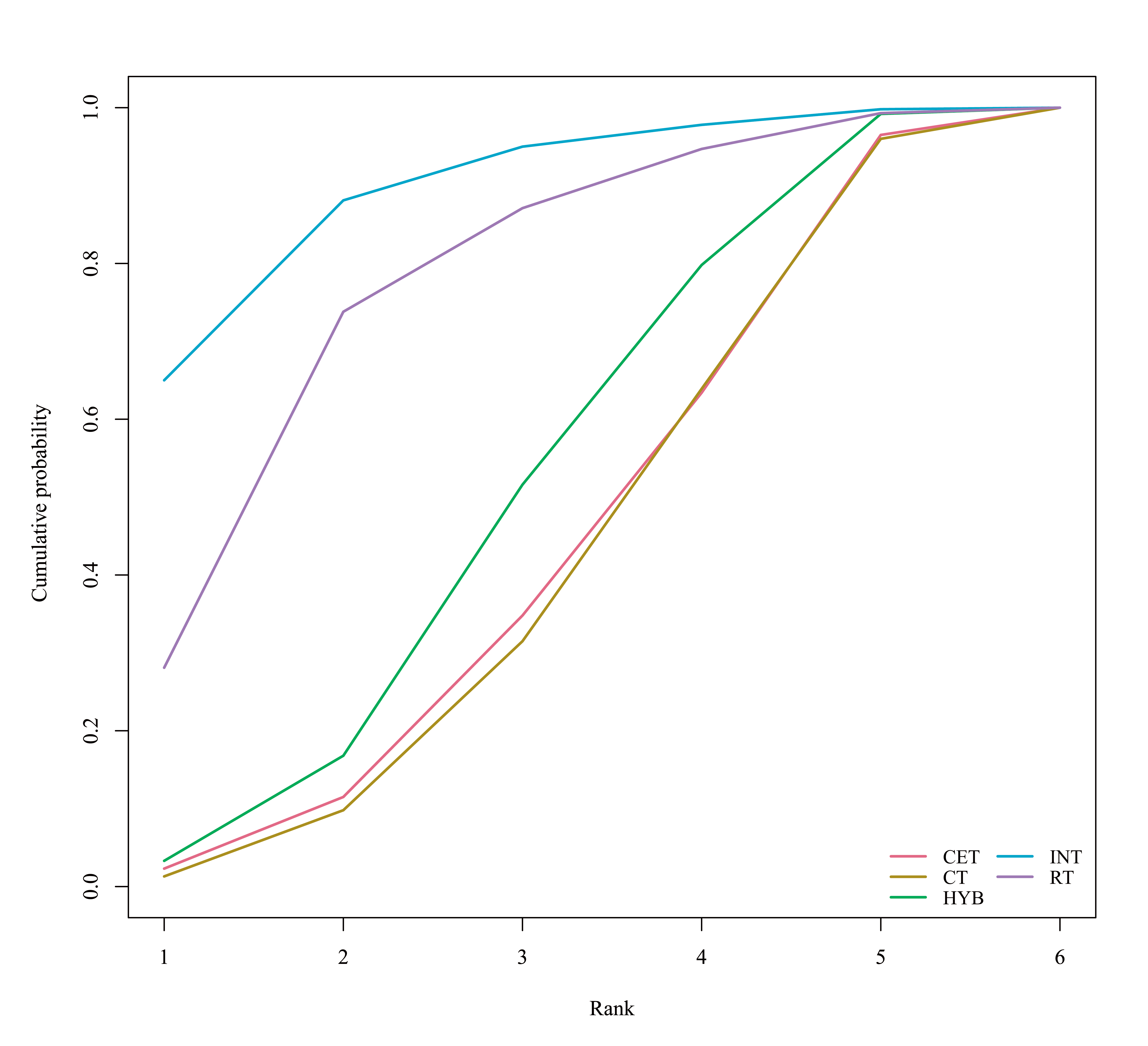
Fig S4.** Cumulative rank probability curves (SUCRA) for ranking uncertainty across exercise modalities for PWV.


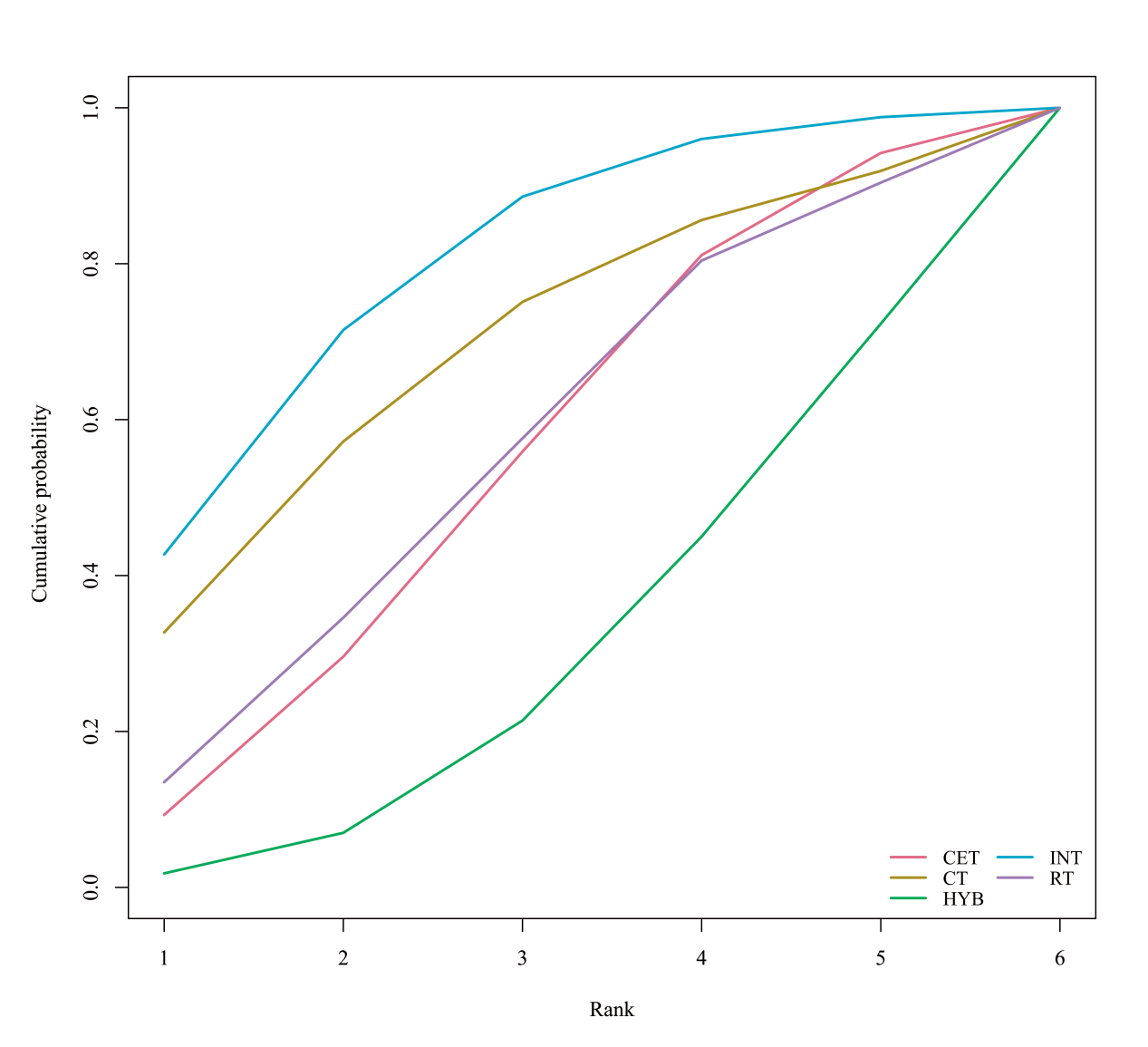


**Fig S5.** Cumulative rank probability curves (SUCRA) for ranking uncertainty across exercise modalities for CIMT.

Curves show the cumulative probability of each modality achieving a given rank or better (Rank 1 = best) based on posterior rank probabilities from the Bayesian random-effects network meta-analysis. SUCRA (0–1) corresponds to the area under each cumulative ranking curve; higher values indicate a higher probability of being among the best-ranked interventions. For PWV and CIMT (where lower values indicate benefit), the ranking direction was defined such that higher ranks/SUCRA correspond to greater improvement (i.e., larger reductions). Substantial overlap among curves indicates uncertainty in treatment ordering; rankings should therefore be interpreted cautiously, particularly where CINeMA confidence is low or very low.
