# Supplementary material for: The effect of exercise intervention on atherosclerosis prevention in overweight or obese adults: A Bayesian network meta-analysis of randomized controlled trials
Source: PLoS One. 2026 Mar 13;21(3):e0344674. doi: 10.1371/journal.pone.0344674 (PMC12987468; doi:10.1371/journal.pone.0344674)
Supplement: S1 Results — (DOCX) [file pone.0344674.s007.docx]

**S1Results. Assessment of risk of bias of randomized trials with the Risk of Bias 2 (RoB2) .**


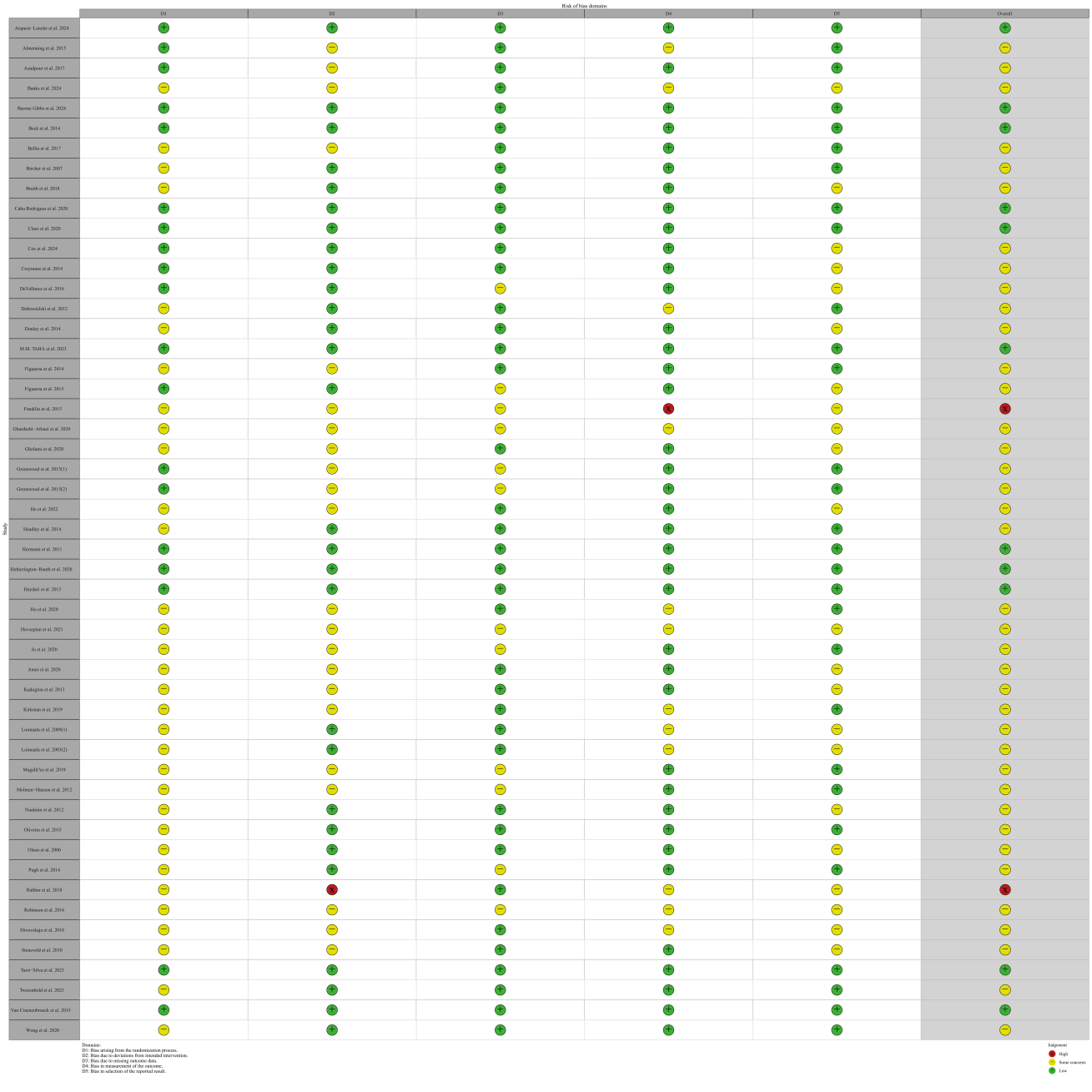


**Fig S1.** Risk of bias assessment for randomized controlled trials (RoB 2).


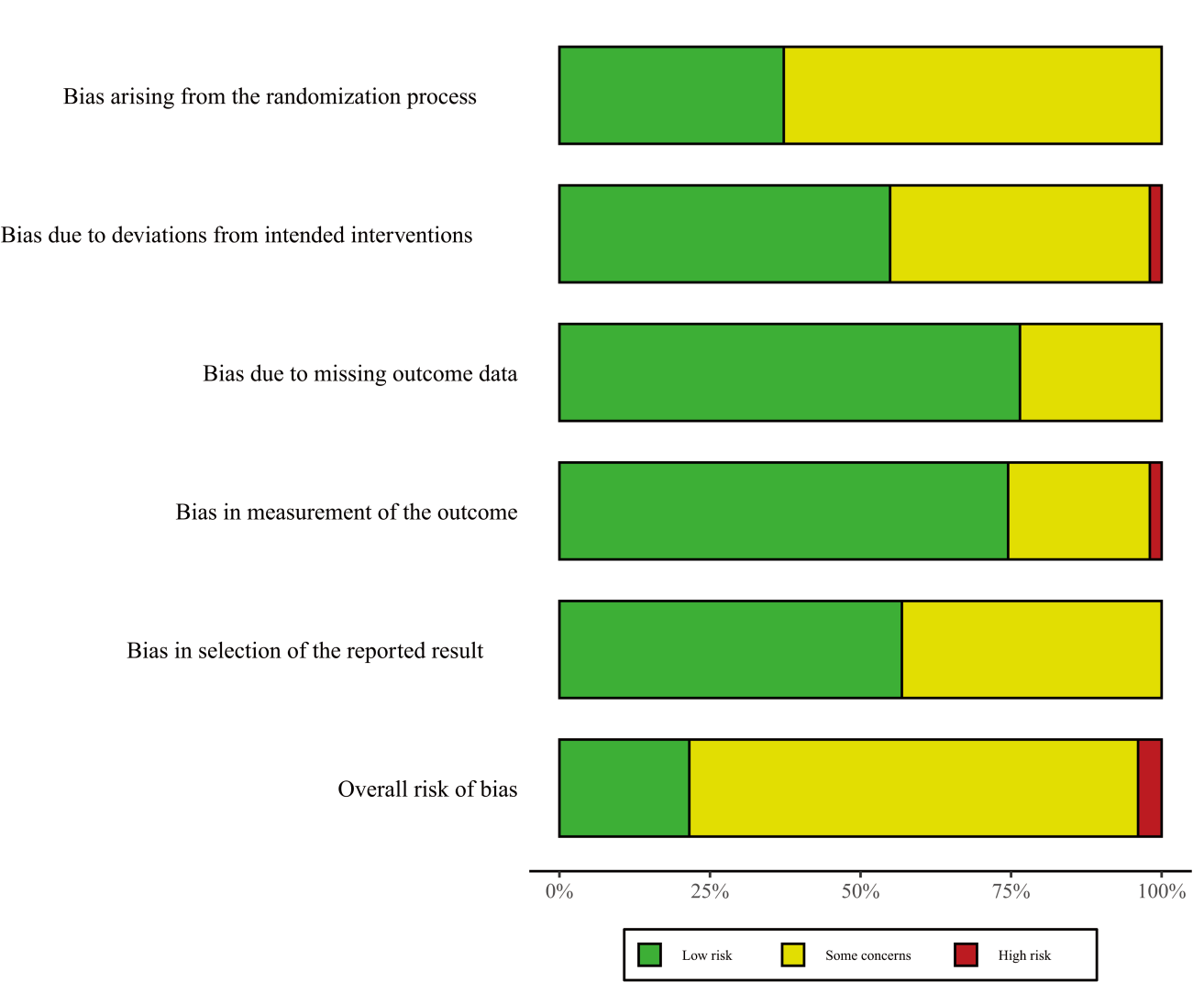


**Fig S2.** Risk of bias assessment for randomized trials (RoB2).
