# Supplementary material for: The effect of exercise intervention on atherosclerosis prevention in overweight or obese adults: A Bayesian network meta-analysis of randomized controlled trials
Source: PLoS One. 2026 Mar 13;21(3):e0344674. doi: 10.1371/journal.pone.0344674 (PMC12987468; doi:10.1371/journal.pone.0344674)
Supplement: S2 File — (DOCX) [file pone.0344674.s009.docx]

**S2 File. Effect size computation and imputation of missing standard deviations**

**Overview**

Primary effect sizes were standardized mean differences (SMDs) computed as Hedges’ g using post-intervention (end-of-treatment) means and SDs for intervention and control arms. This construction does not require assumptions about within-person pre–post correlations. When SDs were not reported but standard errors (SEs) or 95% confidence intervals (CIs) for the post-intervention means were available, SDs were derived using the conversion rules below. Trials for which post-intervention SDs could not be obtained from reported information were not included in the primary effect-size computation for that outcome; such cases, when present, were handled in sensitivity or qualitative synthesis as appropriate.

**A. Hedges’ g from post-intervention means and SDs**

For study i, using end-of-intervention (post-treatment) data, let $n_{1}$, $m_{1}$and $sd_{1}$ denote the sample size, mean, and SD in the intervention arm, and let $n_{2}$, $m_{2}$and $sd_{2}$ denote the corresponding quantities in the control arm.

Pooled SD:

$${SD}_{pooled}=\sqrt{\frac{(n_{1}-1){sd}_{1}^{2}+(n_{2}-1){sd}_{2}^{2}}{n_{1}+n_{2}-2}}$$

Cohen’s d:

$$d=\frac{m_{1}-m_{2}}{{SD}_{pooled}}$$

Small-sample correction (Hedges’ J):
Define the degrees of freedom as:

$$df=n_{1}+n_{2}-2$$

The small-sample correction factor is:

$$J=1-\frac{3}{4df-1}$$

which is algebraically equivalent to:

$$J=1-\frac{3}{4(n_{1}+n_{2})-9}$$

Hedges’ g (SMD):

$$g=J\cdot d$$

**B. Sampling variance and SE of Hedges’ g**

The sampling variance of Hedges’ g was calculated as:

$$v_{g}=\frac{n_{1}+n_{2}}{n_{1}n_{2}}+\frac{g^{2}}{2(n_{1}+n_{2}-2)}$$

The standard error used in meta-analysis was:

$$se_{g}=\sqrt{v_{g}}$$

**C. Deriving SDs from SEs (or SEMs)**

When a study reported an SE (or SEM) for a post-intervention mean, the SD was derived as:

$$SD=SE\cdot\sqrt{n}$$

where n is the sample size for that arm at the post-intervention assessment. If the reported quantity was explicitly labeled SEM, it was treated as SE for this conversion.

**D. Deriving SDs from 95% CIs for post-intervention means**

When a study reported a 95% CI for a post-intervention mean (m) in a given arm, with lower and upper bounds (L, U), we first derived the SE as:

$$SE=\frac{U-L}{2\cdot t_{0.975,df}}$$

where $df=n-1$ and $df$ is the 97.5th percentile of the t distribution with df degrees of freedom. The SD was then obtained via:

$$SD=SE\cdot\sqrt{n}$$

This procedure assumes the reported CI pertains to the mean (not to a mean difference).

**E. Processing workflow and decision rules for missing SDs**

1) Extract post-intervention means and dispersion for each arm (SD preferred; otherwise SE/SEM; otherwise 95% CI for the mean).
2) If SD is missing, derive SD using the conversion rules in Sections C-D.
3) If SD is not reported and cannot be derived from SE/SEM or 95% CI information, the study was not included in the primary post-intervention effect-size calculation for that outcome.
4) Primary effect sizes were computed from post-intervention data; baseline-only or change-score-only reporting was not used in the primary analysis because it would require additional assumptions (e.g., pre-post correlations) or non-comparable variance definitions. Studies reporting change scores only were considered in sensitivity analyses or described qualitatively, as appropriate.
5) All conversions were performed at the arm level using the sample size corresponding to the reported post-intervention summary (accounting for attrition when reported).

**Implementation note**

All effect sizes and sampling variances were implemented using standard meta-analytic routines (e.g., metafor::escalc, measure = "SMD"), with arm-level SDs supplied directly or derived as described above prior to computing Hedges’ g and its sampling variance.
