# Supplementary material for: The effect of exercise intervention on atherosclerosis prevention in overweight or obese adults: A Bayesian network meta-analysis of randomized controlled trials
Source: PLoS One. 2026 Mar 13;21(3):e0344674. doi: 10.1371/journal.pone.0344674 (PMC12987468; doi:10.1371/journal.pone.0344674)
Supplement: S3 File — (DOCX) [file pone.0344674.s010.docx]

**S3 File. Bayesian NMA transparency: model specification, priors, software environment, and MCMC settings**

**A. Data and effect size (SMD)**

For each outcome (FMD, PWV, CIMT), trial arms were summarized on a standardized mean difference (SMD; Hedges’ g) scale. For each study i and each non-baseline arm k (k = 2,…, n_i), we defined an observed contrast y_{ik} comparing arm k to the within-trial baseline arm b_i (typically the control arm when present), with corresponding standard error se_{ik}. These contrasts and standard errors were the inputs to the Bayesian NMA model.

**B. Random-effects consistency model**

We fitted a random-effects consistency model with a normal likelihood and identity link. Let t_{ik} denote the treatment in arm k of trial i, and let d_t be the basic parameter representing the average effect of treatment t relative to the reference treatment (control; t = 1). The underlying (true) study-specific relative effect for arm k versus baseline b_i is denoted by δ_{ik}. The model is:

**Model equations**

Likelihood:
 y_{ik} ~ Normal(δ_{ik}, se_{ik}^2)

Consistency structure:
 δ_{ik} = (d_{t_{ik}} − d_{t_{i b_i}}) + u_{ik}

Random effects:
 u_{ik} ~ Normal(0, τ^2)

where τ is the between-study heterogeneity (common across all comparisons within the same outcome network). The reference treatment is set by d_1 = 0 to ensure identifiability.

**C. Handling multi-arm trials**

Multi-arm trials were modeled jointly to preserve within-trial correlations induced by shared comparators and to avoid double-counting participants. For a trial with n_i arms, the vector of random effects u_i = (u_{i2},…, u_{i n_i}) was modeled with a multivariate normal structure that induces positive correlations between comparisons sharing the same baseline. In practice, we used the standard multi-arm adjustment for random-effects NMA, which is equivalent to assuming a correlation of 0.5 between any pair of within-trial random effects for contrasts that share a common comparator (Lu–Ades framework). This joint modeling ensures correct uncertainty propagation for multi-arm evidence.

**D. Prior distributions**

We used weakly informative priors for treatment effects and an explicit prior for heterogeneity. Priors were specified identically for each outcome unless otherwise stated:

 Basic treatment effects:
 d_t ~ Normal(0, 2^2) for t = 2,…, T (with d_1 = 0)

 Heterogeneity:
 τ ~ Half-Student-t(ν = 3, 0, 0.5) (τ ≥ 0)

These priors constrain implausibly large SMDs while remaining weakly informative for typical effects in exercise intervention trials. Sensitivity checks with alternative weakly informative priors for τ (e.g., half-normal with scale 0.5) yielded materially similar posterior summaries.

**E. Computation and MCMC settings (Stan/brms implementation)**

Models were fitted in R using a Stan-based MCMC engine via brms. We ran 4 independent chains with a prespecified warm-up/burn-in and no thinning. To reduce Monte Carlo error, total iterations were increased beyond 7,000 per chain, and stability of key posterior summaries and ranking outputs was verified. Convergence was assessed using trace plots and quantitative diagnostics (R-hat and effective sample sizes).

**Software environment (Stan/brms)**

Analyses were conducted in R version 4.4.2 (2024-10-31 ucrt) on Windows 11 x64 (build 26200). Bayesian models were fitted using brms 2.22.0 with the rstan 2.32.6 backend (Stan 2.32.2; StanHeaders 2.32.10). Key supporting packages included metafor 4.8.0, netmeta 3.2.0, ggplot2 4.0.0, bayesplot 1.11.1, loo 2.8.0, and posterior 1.6.0.

**F. Additional Bayesian random-effects meta-analysis (JAGS) used for MCMC diagnostic plots**

For certain MCMC diagnostic visualizations (density, trace, autocorrelation, running mean, R-hat, Geweke), we implemented a Bayesian random-effects meta-analysis in JAGS using a normal likelihood on the SMD scale. Let y_i denote the observed SMD for study i with sampling variance v_i. The model is:

 y_i ~ Normal(δ_i, v_i)
 δ_i ~ Normal(d, τ^2)
 d ~ Normal(0, 1.0E-5)
 τ ~ Uniform(0, 5)

This specification corresponds to a conventional Bayesian random-effects meta-analysis for a single overall effect d with heterogeneity τ.

**Software environment (JAGS)**

Analyses were conducted in R 4.4.2 using R2jags 0.8.9 and rjags 4.16 connected to JAGS 4.3.1. MCMC diagnostics and visualizations were generated using ggmcmc 1.5.1.1 and mcmcplots 0.4.3.

**MCMC settings (JAGS)**

We ran 3 chains with 50,000 iterations per chain, including 20,000 burn-in iterations and thinning = 10, yielding 3,000 post-burn-in posterior draws per chain (total 9,000 draws across chains). Convergence was assessed using trace plots and quantitative diagnostics including R-hat and Geweke statistics.
